# Actin limits egg aneuploidies associated with female reproductive aging

**DOI:** 10.1126/sciadv.adc9161

**Published:** 2023-01-20

**Authors:** Sam Dunkley, Binyam Mogessie

**Affiliations:** ^1^ School of Biochemistry, University of Bristol, Bristol BS8 1TD, UK.; ^2^ Department of Molecular, Cellular, and Developmental Biology, Yale University, New Haven, CT 06511, USA.

## Abstract

Aging-related centromeric cohesion loss underlies premature separation of sister chromatids and egg aneuploidy in reproductively older females. Here, we show that F-actin maintains chromatid association after cohesion deterioration in aged eggs. F-actin disruption in aged mouse eggs exacerbated untimely dissociation of sister chromatids, while its removal in young eggs induced extensive chromatid separation events generally only seen in advanced reproductive ages. In young eggs containing experimentally reduced cohesion, F-actin removal accelerated premature splitting and scattering of sister chromatids in a microtubule dynamics–dependent manner, suggesting that actin counteracts chromatid-pulling spindle forces. Consistently, F-actin stabilization restricted scattering of unpaired chromatids generated by complete degradation of centromeric cohesion proteins. We conclude that actin mitigates egg aneuploidies arising from age-related cohesion depletion by limiting microtubule-driven separation and dispersion of sister chromatids. This is supported by our finding that spindle-associated F-actin structures are disrupted in eggs of reproductively older females.

## Teaser

Actin counteracts microtubule-based pulling forces to reduce the effects of chromosome cohesion loss in aged mammalian eggs.

## INTRODUCTION

Healthy embryo formation and development critically hinges on the accuracy of meiotic chromosome segregation. When mammalian eggs are formed from oocytes, a meiotic spindle machinery composed of the microtubule and actin cytoskeletons separates the chromosomes (*1*). Until this programmed separation occurs, it is crucial that oocyte-homologous chromosomes, each containing a pair of sister chromatids, are tightly held together in a bivalent structure (*2*). This association is predominantly achieved by cohesin, a multisubunit protein complex that links chromosome arms and the centromeres of sister chromatids (*2*–*4*). During oocyte meiosis I, cohesin is removed from chromosome arms by Separase-mediated cleavage of its meiosis-specific component Rec8 (*2*, *5*–*7*). Simultaneously, cohesin between sister chromatid centromeres is protected by Shugoshin2 shielding Rec8 from Separase activity (*8*–*10*). When coupled with microtubule-based pulling forces that are exerted on the chromosomes’ kinetochores (*1*, *11*, *12*), this localized cohesion protection provides oocytes with a mechanism to selectively separate homologs at anaphase I without disrupting sister chromatid linkage. In the newly formed egg, centromeric cohesion is maintained between the sister chromatids, which are attached to and pulled by kinetochore microtubule fibers originating from opposite spindle poles (*1*, *2*). This tug-of-war in which microtubule-based pulling forces are resisted by sister centromere cohesion effectively generates tension between the sister chromatids’ kinetochores (*13*). Unprogrammed centromeric cohesion loss in eggs can therefore cause premature separation of sister chromatids by kinetochore-bound microtubules and increases their likelihood of missegregation (*2*, *14*–*16*).

The incidence of chromosome segregation errors and egg aneuploidy increases with advancing maternal age (*2*, *17*). Because female reproductive aging is also associated with gradual cohesin depletion in oocytes (*2*, *18*, *19*), premature cohesion loss is considered a major cause of egg aneuploidies broadly associated with human infertility and genetic disorders (*2*, *14*, *17*, *20*). However, this progressive cohesin loss does not satisfactorily explain the almost exponential increase in egg aneuploidy that is observed near the end of female reproductive life (*17*, *20*). Because the actin cytoskeleton is a meiosis-specific functional component of the mammalian chromosome segregation machinery (*1*, *21*–*23*), we have here addressed whether its dysfunction in eggs could explain this notable reproductive aging phenomenon.

## RESULTS

### Spindle F-actin is disrupted in reproductively older females

**Fig. 1.**
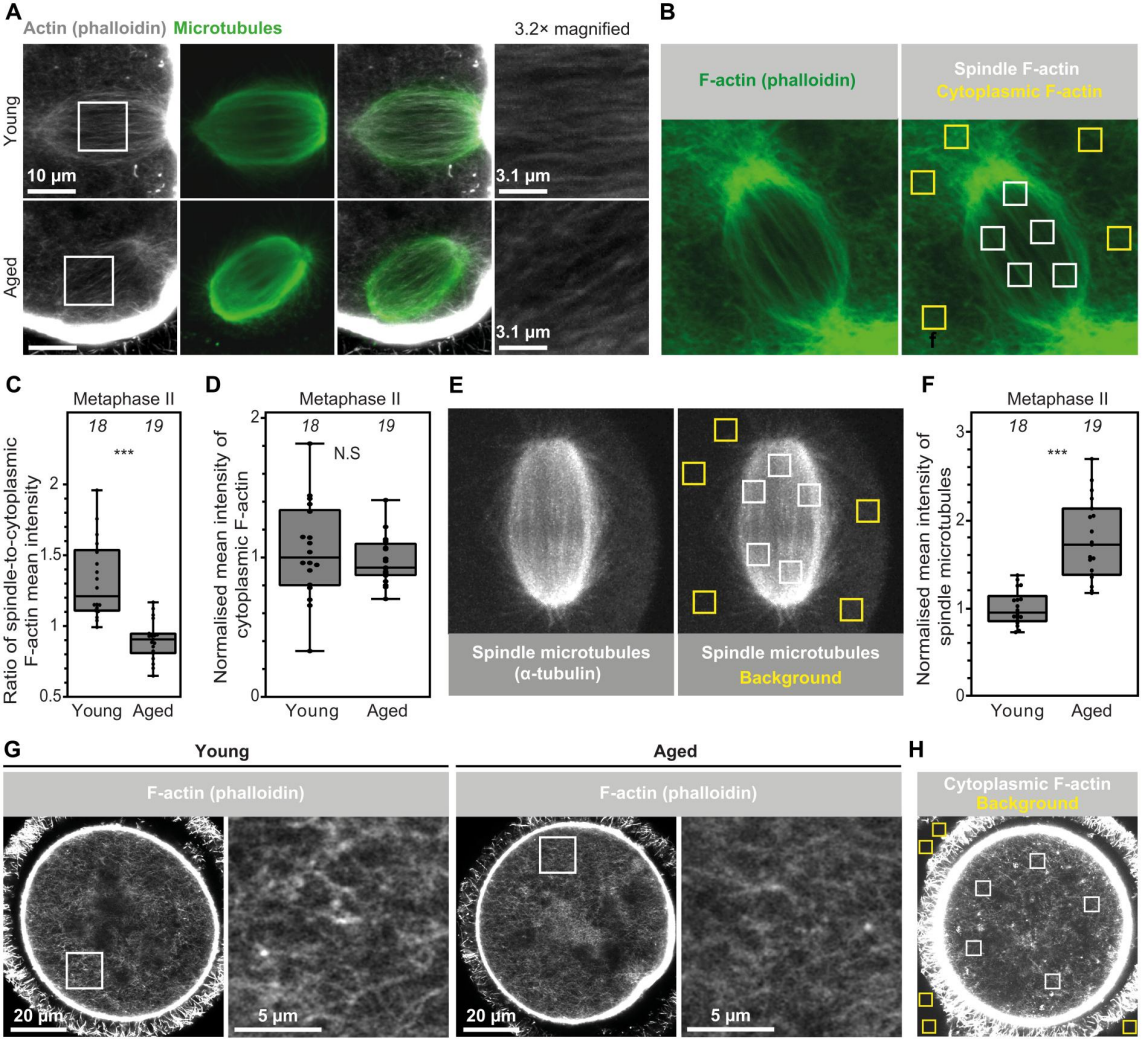
Spindle F-actin is disrupted during female reproductive aging. (**A**) Sum intensity projections of phalloidin-labeled spindle F-actin and microtubules in young and aged metaphase II–arrested eggs. Boxes mark regions that are magnified in insets. (**B**) Method (described in Materials and Methods) for quantification of spindle-to-cytoplasmic F-actin mean fluorescence intensity ratio in metaphase II–arrested eggs of reproductively young or aged mice. (**C**) Quantification of ratio of spindle-to-cytoplasmic F-actin mean fluorescence intensity in young and aged metaphase II–arrested eggs. Data are from three independent experiments. N.S., not significant *** *P*<0.0001. (**D**) Normalized cytoplasmic F-actin mean fluorescence intensities in young and aged metaphase II–arrested eggs. Data are from three independent experiments. (**E**) Method (described in Materials and Methods) for quantification, background correction, and normalization of spindle microtubule mean fluorescence intensities in eggs of reproductively young or aged mice. (**F**) Normalized spindle microtubule mean fluorescence intensities in young and aged metaphase II–arrested eggs. Data are from three independent experiments. (**G**) Representative single section Airyscan images of phalloidin-labeled cytoplasmic F-actin structures in metaphase II–arrested eggs of reproductively young or aged mice. Boxes mark regions that are magnified in insets. (**H**) Method (described in Materials and Methods) for quantification, background correction, and normalization of cytoplasmic F-actin mean fluorescence intensities in eggs of reproductively young or aged mice. Statistical significance was evaluated using Mann-Whitney *t* test (C, D, and F). The number of analyzed oocytes is specified in italics.

To examine the impact of reproductive aging on cellular F-Actin integrity, we first applied quantitative superresolution microscopy and visualized fluorescent phalloidin-labeled (*22*) actin structures in metaphase II–arrested eggs developed from oocytes of reproductively young (6 to 12 weeks old) and aged (58 to 62 weeks old) mice. These imaging data showed that spindle F-actin in aged eggs appeared reduced and disorganized (Fig. 1A). To quantify this change, we measured fluorescence intensity ratios between several randomly selected regions containing spindle F-actin structures and cytoplasmic F-actin networks (Fig. 1B). This revealed that spindle F-actin fluorescence intensity is significantly reduced in aged eggs (Fig. 1C) This analysis further showed that cytoplasmic F-actin network density was not disrupted in aged eggs (Fig. 1D). Female reproductive aging is therefore accompanied by meiotic spindle-specific disruption of F-actin in mammalian eggs. We previously showed that spindle F-actin assembly in mouse oocytes is dependent on integrity of spindle microtubules (*21*). To test whether age-related spindle F-actin reduction arises from microtubule disruption, we performed microtubule fluorescence intensity measurements in metaphase II spindles of young and aged eggs (Fig. 1E). Unexpectedly, this analysis showed that meiotic spindles in aged eggs contain significantly increased microtubule intensity (Fig. 1F). Therefore, we conclude that reproductive age-related disruption of spindle F-actin is not caused by diminished meiotic spindle microtubule content in aged eggs.

### Actin disruption exacerbates premature chromatid separation in aged eggs

**Fig. 2.**
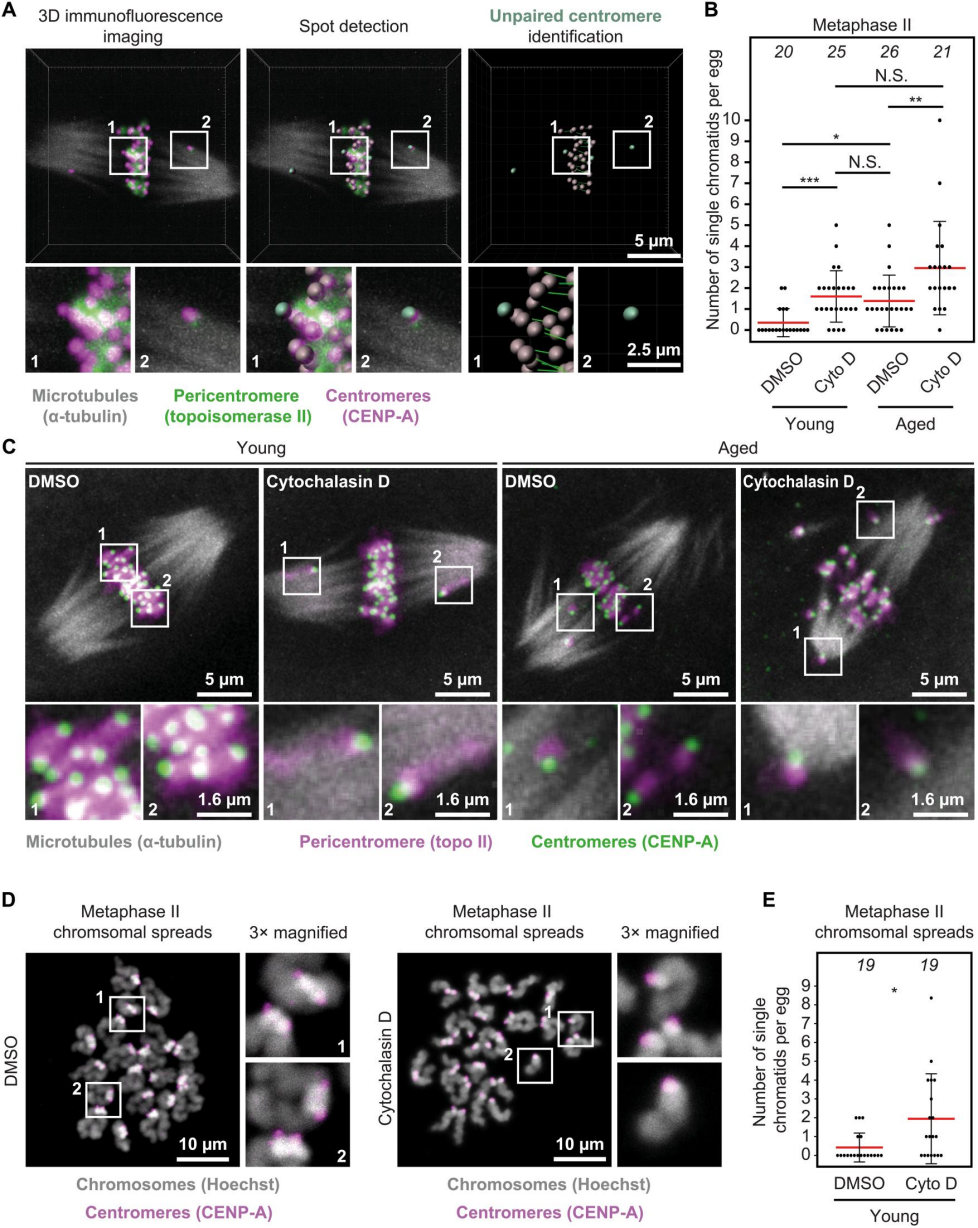
F-actin loss exacerbates reproductive age-related premature chromatid separation in mammalian eggs. (**A**) Unpaired chromatid identification pipeline from maximum intensity projected immunofluorescence images of CENP-A, topoisomerase II, and microtubules in young and aged metaphase II–arrested eggs. CENP-A spots detection and intercentromere distance measurements in Imaris were used to identify chromatids that did not contain two centromeres and assign them as single chromatids. Boxes mark regions that are magnified in insets. (**B**) Quantification of the number of single chromatids [identified as in (C)] in DMSO- or cytochalasin D–treated young and aged metaphase II–arrested eggs. Each filled black circle in graph represents a single egg, and red bars represent mean values. Data are from three independent experiments. (**C**) Representative maximum intensity projected immunofluorescence images of microtubules, centromeres, chromatid pairs, and single chromatids in DMSO- or cytochalasin D–treated young and aged metaphase II–arrested eggs. Boxes mark regions that are magnified in insets. (**D**) Representative maximum intensity projected immunofluorescence images of centromeres and chromatids in metaphase II chromosomal spreads of DMSO- or cytochalasin D–treated young eggs. Boxes mark regions that are magnified in insets. (**E**) Quantification of the number of single chromatids in metaphase II chromosomal spreads of DMSO- or cytochalasin D–treated young eggs. Each filled black circle in graph represents chromosomal spread from a single egg, and red bars represent mean values. Data are from three independent experiments. Statistical significance was evaluated using Fisher’s exact test (B and E) * *P*=0.0131. The number of analyzed oocytes is specified in italics. Cyto D, cytochalasin D.

We next addressed whether F-actin disruption contributes to egg aneuploidy in reproductively older females. Here, we first visualized meiotic spindles, chromosomes, and centromeres in metaphase II–arrested mouse eggs using high-resolution three-dimensional (3D) immunofluorescence microscopy. We then identified sister centromere pairs using automated spot detection in Imaris software (Fig. 2A) and counted prematurely separated chromatids. This approach revealed that 10 of 26 of dimethyl sulfoxide (DMSO)–treated control eggs from reproductively older (35 to 39 weeks old) females had at least two prematurely separated chromatids (Fig. 2, B and C). This observation is consistent with high incidence of aneuploidy in aged eggs that generally arises from centromeric cohesion loss (*2*, *15*, *18*, *19*). By stark contrast, cytochalasin D–mediated disruption of F-actin (*21*, *24*) significantly increased the incidence of untimely chromatid separation in 17 of 21 of aged eggs (Fig. 2, B and C). These data strongly argue that the integrity of the actin cytoskeleton critically limits the extent of premature chromatid separation arising from age-related centromeric cohesion loss.

### Actin disruption causes aging-like chromatid disengagement in young eggs

To better understand the contribution of actin to chromosome organization in female meiosis, we disrupted F-actin structures in eggs isolated from reproductively young (6 to 12 weeks old) mice. Compared to 2 of 20 of DMSO-treated control young eggs, 12 of 25 of cytochalasin D–treated young eggs displayed at least two prematurely separated chromatids (Fig. 2, B and C), which is notably comparable to incidence of chromatid separation in control aged eggs (Fig. 2B). We were able to reproduce this high incidence of centromeric cohesion loss by treating metaphase II–arrested young eggs with latrunculin B, a mechanistically distinct F-actin–disrupting compound (fig. S1A) (*24*–*27*). To independently confirm these observations, we performed chromosome spreads of metaphase II–arrested DMSO- or cytochalasin D–treated young eggs. We then combined 3D immunofluorescence microscopy of chromatids and centromeres (Fig. 2D) with spot detection algorithms in Imaris software to identify prematurely separated chromatids (fig. S1B). Consistent with our data obtained from intact oocytes, this chromosome spread approach revealed that incidence of untimely chromatid splitting is significantly increased when F-actin is disrupted with cytochalasin D (Fig. 2, D and E) or latrunculin B (fig. S1, C and D).

During metaphase II, microtubule-mediated pulling of sister chromatids generates tension between their centromeres. We therefore predicted that high incidence of premature chromatid separation in F-actin–disrupted eggs is accompanied by increased tension and thus wider spacing between sister centromeres. To test this, we took further advantage of our quantitative immunofluorescence microscopy assays and measured intercentromere distances in DMSO- or cytochalasin D–treated intact eggs and chromosomal spreads (fig. S1, E and F). These measurements did not show increased intercentromere tension after F-actin disruption (fig. S1, G and H). Because this analysis only includes centromeres of unseparated chromatids, it is likely that inclusion of prematurely separated centromere pairs, which are generally spaced several micrometers apart (Fig. 2, A, C, and D), would reveal significant differences in intercentromere distance after F-actin disruption. Normal spacing between unseparated centromeres (fig. S1, G and H) suggests that removal of F-actin may rather predispose only a subset of chromatid pairs to untimely separation.

To understand how F-actin disruption promotes uncontrolled chromatid separation, we asked whether the cohesin complex is compromised in cells devoid of F-actin. To address this, we performed high-resolution immunofluorescence microscopy of endogenous Rec8 in oocytes wherein cohesion proteins can be more readily visualized along chromosome arms (fig. S2A). We then used an unbiased image analysis pipeline in Imaris software to automatically identify chromosomal surfaces in 3D and quantified within these volumes the fluorescence intensity of Rec8 (fig. S2B). This analysis revealed that cytochalasin D treatment did not reduce Rec8 protein abundance between chromosomes (fig. S2, C to E, and movies S1 and S2). Further quantification of Rec8 immunofluorescence intensity in chromosomal spreads (fig. S2, F and G) of DMSO- or cytochalasin D–treated oocytes confirmed this result (fig. S2, H and I). Further immunofluorescence analysis showed significant reduction of Rec8 protein in Monopolar spindle 1 (MPS1) kinase inhibitor reversine-treated oocytes that are defective in centromeric cohesion (*28*) (fig. S3, A to D). This demonstrates that our quantitative microscopy assay of Rec8 abundance is suitable for detection of changes in cohesion protein level that are sufficient to disrupt sister chromatid association. It is therefore unlikely that canonical chromosome cohesion pathways are affected in F-actin–disrupted eggs that display extensive premature sister chromatid separation. Collectively, our data demonstrate that removal of F-actin in young eggs is sufficient to induce aging-like chromatid separation events that underlie high incidence of egg aneuploidy at later reproductive ages.

### Actin disruption accelerates chromatid separation following cohesion loss

**Fig. 3.**
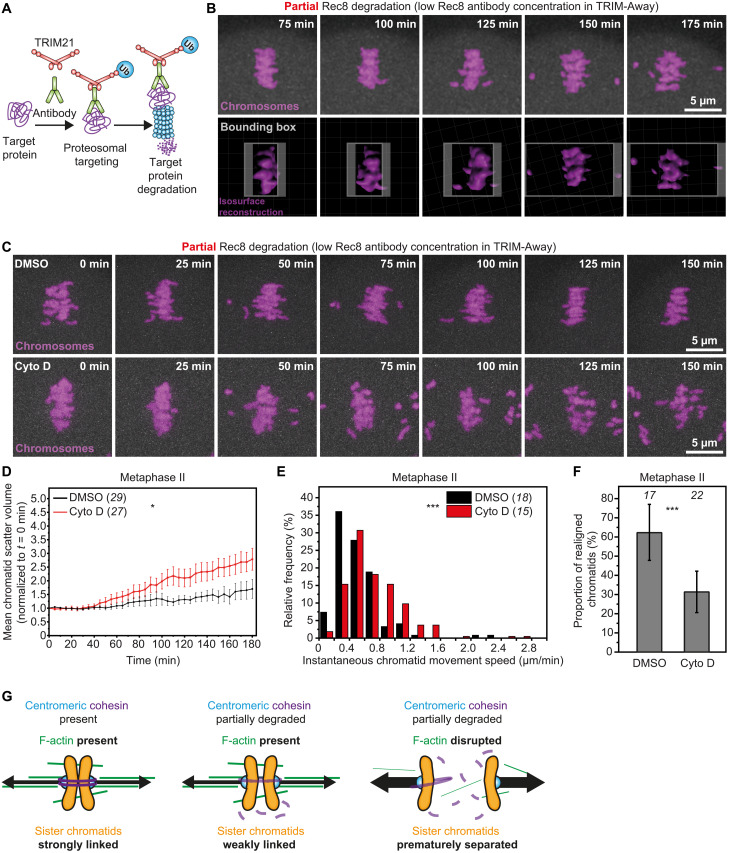
F-actin disruption accelerates aging-like premature chromatid separation in young eggs. (**A**) The TRIM-Away system entails microinjection of antibodies against a protein of interest and transient expression of the E3 ubiquitin ligase TRIM21, which binds the Fc domain of antibodies with high affinity. TRIM21 then recruits antibody-bound target proteins for degradation by the proteasome. (**B**) Stills from time-lapse movie of chromosomes (H2B-mRFP, top) and isosurface reconstructions (bottom) in a metaphase II–arrested egg with partially degraded Rec8. Pseudo object-orientated bounding box (gray, bottom) showing progressive increase in the minimal cuboid volume that encloses all chromatids as a measure of chromatid scattering. (**C**) Stills from representative time-lapse movies of chromosomes (H2B-mRFP) in DMSO- or cytochalasin D–treated metaphase II–arrested eggs with partially degraded Rec8. (**D**) Normalized chromatid scatter volumes measured as in (B) over 3 hours in DMSO- or cytochalasin D–treated metaphase II–arrested eggs with partially degraded Rec8. Black and red lines represent mean values. Error bars represent SEM. Nonaveraged individual measurements are provided in fig. S5 (A and B) * *P* = 0.0300. Data are from three independent experiments. (**E**) Distribution of instantaneous chromatid movement speeds in DMSO- or cytochalasin D–treated metaphase II–arrested eggs with partially degraded Rec8. Data are from three independent experiments *** *P*<0.0001. (**F**) Proportion of scattered chromatids that reestablished alignment to the spindle equator in DMSO- or cytochalasin D–treated metaphase II–arrested eggs with partially degraded Rec8. Error bars represent SD. Data are from three independent experiments *** P<0.0001. (**G**) Graphical representation of the effect of F-actin loss on chromatid linkage in eggs containing partially reduced centromeric cohesion. Statistical significance was evaluated using two-way ANOVA (D), two-tailed Student’s *t* test (E), or Fisher’s exact test (F). The number of analyzed oocytes is specified in brackets and in italics. Cyto D, cytochalasin D.

To uncover how F-actin disruption accelerates chromatid separation, we first sought to develop an experimental system in which centromeric cohesion and cytoskeletal dynamics can be manipulated simultaneously. Reasoning that experimentally induced depletion of cohesin might mimic the maternal aging effect of premature chromatid separation in young eggs, we implemented TRIM-Away—a recently developed method in which the ubiquitin ligase Tripartite Motif Containing 21 (TRIM21) targets antibody-bound endogenous proteins of interest for degradation via the proteosome (Fig. 3A) (*29*)—to acutely disrupt centromeric cohesion. TRIM-Away is a well-established tool for rapid protein degradation in mammalian oocytes (*29*, *30*), embryos (*31*), as well as in other vertebrate and invertebrate models (*32*–*36*). To induce aging-like sister chromatid separation via this approach, we microinjected control immunoglobulin Gs (IgGs) or low concentrations of Rec8 heavy-chain antibodies (to only partially degrade Rec8) into TRIM21-expressing, metaphase II–arrested young eggs. High-resolution live imaging of chromatids (marked with histone H2B-monomeric red fluorescent protein (mRFP) at 5-min intervals showed that partial Rec8 depletion in this experimental system caused modest separation and dispersion of sister chromatids (Fig. 3B and movie S3). To quantify this, we reconstructed the 3D volumes of chromatids (movie S4) in Imaris software, performed object-oriented bounding box analysis, and measured the minimal cuboid volume that contained all chromatids at each live-imaging time point (Fig. 3B and fig. S4A). We then plotted this volume measurement over a 3-hour observation time as readout for premature separation and scattering of sister chromatids. This analysis showed that partial Rec8 degradation in DMSO-treated control eggs caused gradual sister chromatid separation with most single chromatids remaining near the spindle equator during the 3-hour observation time (Fig. 3, C and D; fig. S5, A and B; and movie S5) and with some dispersed chromatids often reestablishing alignment with the main chromosome mass (movie S6). By contrast, partial cohesion removal after F-actin disruption markedly accelerated chromatid separation and caused persistent scattering of single chromatids in cytochalasin D–treated eggs (Fig. 3, C and D; fig. S5, A and B; and movies S7 and S8). This finding demonstrated that F-actin disruption exacerbates untimely chromatid separation arising from aging-like centromeric cohesion depletion.

To test the effect of F-actin loss on unprogrammed chromatid splitting dynamics, we tracked prematurely separated chromatids in 3D by high temporal resolution live imaging and measured their frame-to-frame displacement away from the metaphase II spindle equator. This analysis showed that most prematurely separated chromatids in cytochalasin D–treated eggs migrated away from the spindle equator at significantly faster instantaneous speeds than in DMSO-treated control eggs that contained F-actin (Fig. 3E and fig. S6A). This is consistent with a greater effect of chromatid-pulling microtubule forces when F-actin is disrupted. Our 3D chromatid tracking approach also successfully captured distinct realignment events where previously misaligned chromatids were transported back to the spindle equator (fig. S4B). These correction events were common in control eggs: ~62% of misaligned chromatids were repositioned at the spindle equator within the 3-hour observation period (Fig. 3F). By contrast, chromatid realignment was significantly less frequent in cytochalasin D–treated eggs with only ~31% of previously scattered chromatids reestablishing correct alignment at the spindle equator (Fig. 3F). Persistently misaligned chromatids are more likely to be missegregated in anaphase II (*21*). We thus conclude that the actin cytoskeleton plays a critical, two-pronged role in the prevention of chromatid scattering and ultimately egg aneuploidy (Fig. 3G): first, it acts as a brake against accelerated separation of sister chromatids; second, it promotes the realignment of scattered single chromatids.

### Actin stabilization restricts chromatid separation in the absence of cohesion

**Fig. 4.**
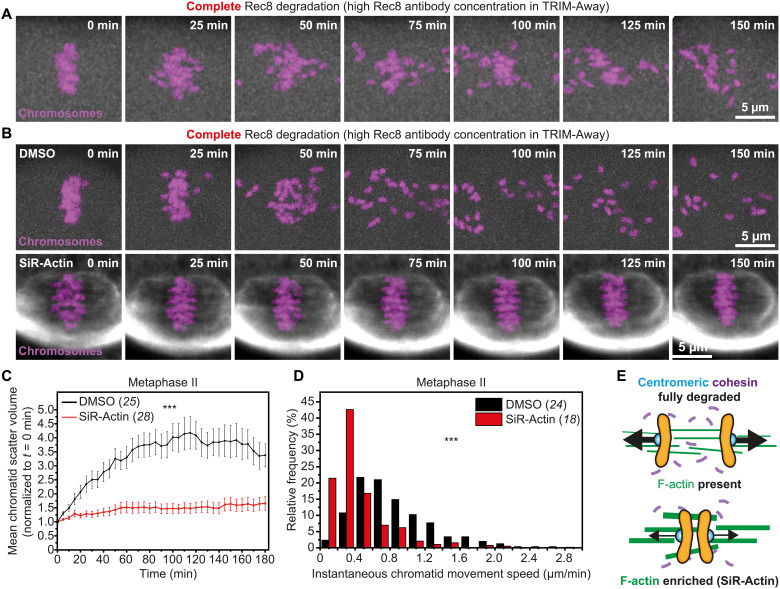
F-actin stabilization blocks premature chromatid separation in the absence of centromeric cohesion. (**A**) Stills from time-lapse movie of chromosomes (H2B-mRFP) in a metaphase II–arrested egg with fully degraded Rec8. (**B**) Stills from representative time lapse movies of chromosomes (H2B-mRFP) in DMSO- or SiR-Actin–treated (fluorescent F-actin shown in gray) metaphase II–arrested eggs with fully degraded Rec8. (**C **) Normalized chromatid scatter volumes measured as in Fig. 2B over 3 hours in DMSO- or SiR-Actin–treated metaphase II–arrested eggs with fully degraded Rec8. Black and red lines represent mean values. Error bars represent SEM. Nonaveraged individual measurements are provided in fig. S5 (C and D) ***P<0.0001. Data are from three independent experiments. (**D**) Distribution of instantaneous chromatid movement speeds in DMSO- or SiR-Actin–treated metaphase II–arrested eggs with fully degraded Rec8. Data are from three independent experiments. (**E**) Graphical representation of the effect of F-actin stabilization on chromatid linkage in eggs with fully disrupted centromeric cohesion. Statistical significance was evaluated using two-way ANOVA (C) or two-tailed Student’s *t* test (D). The number of analyzed oocytes is specified in brackets and in italics.

Having established that F-actin disruption predisposes weakly linked sister chromatids to extensive separation (Fig. 2B), we tested whether its enrichment could help to keep them together in cohesin-deficient eggs. We first stabilized F-actin in young eggs using high concentrations of silicon rhodamine (SiR)-Actin (*21*, *22*), a fluorescence-labeled derivative of Jasplakinolide (*37*) that marks the actin cytoskeleton in living cells (*38*). We then fully degraded centromeric cohesin using highly concentrated Rec8 antibodies in our TRIM-Away strategy (Fig. 4A and movie S9). In DMSO-treated control eggs, this caused extensive separation and scattering of sister chromatids (Fig. 4, B and C; fig. S4C; and movie S10), which is consistent with substantial depletion of cohesin from centromeric regions. In comparison, despite the complete removal of centromeric cohesion, sister chromatids in SiR-Actin–treated eggs largely remained near the spindle equator during 3 hours of observation (Fig. 4, B and C; fig. S5, C and D; and movie S11). Supporting our earlier conclusion that F-actin might act as a brake against their accelerated separation, most poleward migrating chromatids also moved at significantly slower speeds when F-actin was stabilized (Fig. 4D and fig. S6B). These data therefore collectively define a new function of F-actin in restricting the poleward movement of prematurely separated chromatids in eggs with reduced centromeric cohesion (Fig. 4E).

### Dynamic microtubules drive F-actin and cohesion loss-induced chromatid separation

**Fig. 5.**
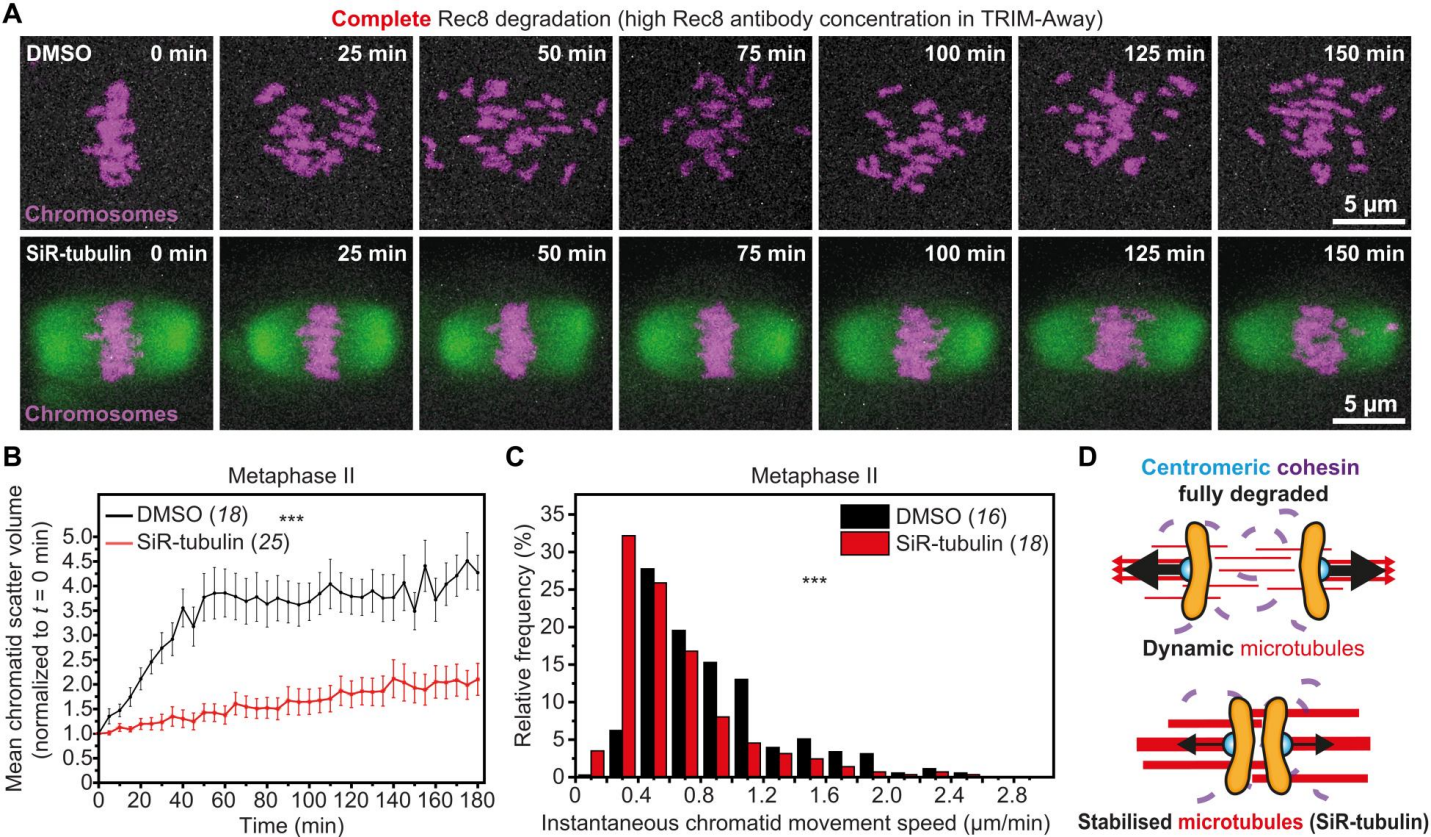
Premature chromatid separation in centromeric cohesion depleted eggs is driven by microtubule dynamics. (**A**) Stills from representative time-lapse movies of chromosomes (H2B-mRFP) in DMSO- or SiR-tubulin–treated (fluorescent microtubules shown in green) metaphase II–arrested eggs with fully degraded Rec8. (**B**) Normalized chromatid scatter volumes measured as in Fig. 2B over 3 hours in DMSO- or SiR-tubulin–treated metaphase II–arrested eggs with fully degraded Rec8. Black and red lines represent mean values. Error bars represent SEM. Nonaveraged individual measurements are provided in fig. S5 (E and F) ***P<0.0001. Data are from three independent experiments. (**C**) Distribution of instantaneous chromatid movement speeds in DMSO- or SiR-tubulin–treated metaphase II–arrested eggs with fully degraded Rec8. Data are from three independent experiments. ***P<0.0001 (**D**) Graphical representation of the effect of blocking microtubule dynamics on chromatid linkage in eggs with fully disrupted centromeric cohesion. Statistical significance was evaluated using two-way ANOVA (B) or two-tailed Student’s *t* test (C). The number of analyzed oocytes is specified in brackets and in italics.

Microtubules are well-established drivers of chromosomal separation during cell division (*1*, *12*). Linking a decline in microtubule function with egg aneuploidy, defective microtubule dynamics were recently found to contribute to chromosomal abnormalities in aged eggs (*39*). We therefore asked whether microtubule dynamics also power chromatid movement in our experimental system of robust cohesion disruption. Consistent with earlier results, TRIM-Away–mediated acute depletion of Rec8 in DMSO-treated control eggs caused extensive dispersion of separated chromatids throughout the meiotic spindle (Fig. 5A and movie S12). This was confirmed by bounding box analyses of chromatid mobility (Fig. 5, B and C; fig. S5, E and F; and fig. S6C). In contrast, blocking of microtubule dynamics before Rec8 degradation by treating eggs with high concentrations of SiR-tubulin—a cell permeable and fluorogenic derivative of the microtubule stabilizing drug docetaxel (*38*, *40*)—significantly slowed down the movement of separated chromatids and restricted their mobility to only near the spindle equator (Fig. 5, B and C; figs. S5, E and F, and S6C; and movie S13). These results demonstrated that microtubule dynamics drive the complete disengagement and scattering of sister chromatids following centromeric cohesion loss (Fig. 5D).

**Fig. 6.**
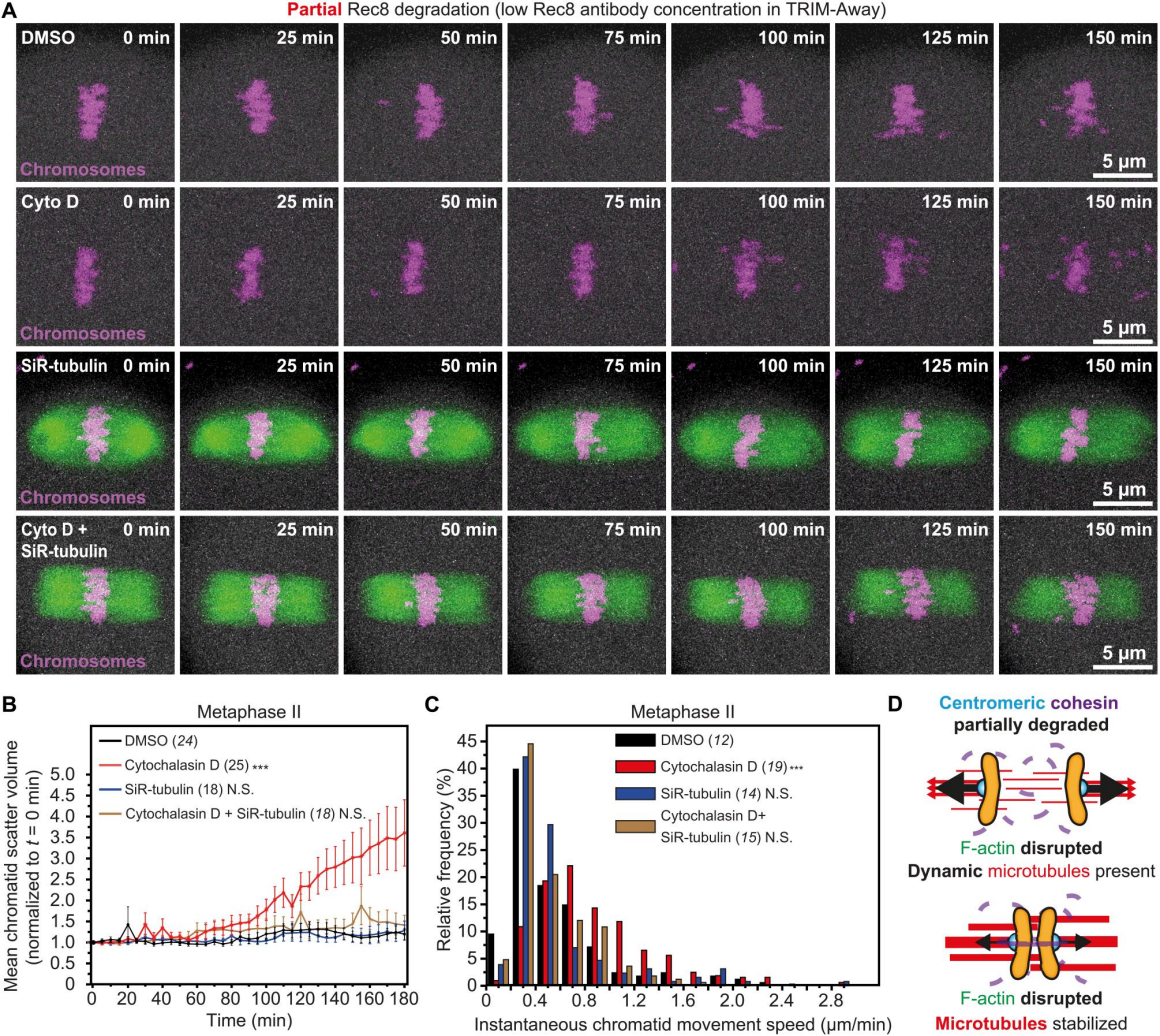
Dynamic microtubules drive F-actin and cohesion loss-induced chromatid separation. (**A**) Stills from representative time lapse movies of chromosomes (H2B-mRFP) in DMSO-, cytochalasin D–, SiR-tubulin– (fluorescent microtubules shown in green), or cytochalasin D and SiR-tubulin–treated metaphase II–arrested eggs with partially degraded Rec8. (**B**) Normalized chromatid scatter volumes measured as in Fig. 2B over 3 hours in DMSO-, cytochalasin D–, SiR-tubulin–, or cytochalasin D and SiR-tubulin–treated metaphase II–arrested eggs with partially degraded Rec8. Black, red, blue, and brown lines represent mean values. Error bars represent SEM. Nonaveraged individual measurements are provided in fig. S5 (G and H) ***P<0.0001. Data are from three independent experiments. (**C**) Distribution of instantaneous chromatid movement speeds in DMSO-, cytochalasin D–, SiR-tubulin–, or cytochalasin D and SiR-tubulin–treated metaphase II–arrested eggs with partially degraded Rec8. Data are from three independent experiments ***P<0.0001. (**D**) Graphical representation of the effect of blocking microtubule dynamics on chromatid linkage in eggs devoid of F-actin and containing partially reduced centromeric cohesion. Statistical significance was evaluated using two-way ANOVA (B) or two-tailed Student’s *t* test (C). The number of analyzed oocytes is specified in brackets and in italics. Cyto D, cytochalasin D.

Spindle F-actin abundance directly affects microtubule organization and dynamics in mouse eggs (*21*). To investigate whether more effective microtubule pulling in the absence of F-actin underpins chromatid separation in cohesion-deficient eggs, we combined cytochalasin D and SiR-tubulin compounds in our experimental system of reproductive aging-like centromeric cohesion depletion (Fig. 3, A and B). In line with our earlier data (Fig. 3, C to E), TRIM-Away–mediated partial Rec8 degradation caused rapid and extensive chromatid scattering only in eggs that did not contain F-actin (Fig. 6, A to C; figs. S5, G and H, and S6D; and movies S14 and S15). Consistent with results showing that partial depletion of Rec8 causes only modest events of chromatid scattering (Fig. 3, C to E), SiR-tubulin–mediated blocking of microtubule dynamics in these eggs had negligible effect on the dispersion of prematurely separated chromatids (Fig. 6, A and B; fig. S5, G and H; and movie S16). Similarly, the movement of unpaired chromatids was unaffected when microtubules were stabilized in eggs that were partially depleted of Rec8 (Fig. 6C and fig. S6D). In notable contrast, blocking microtubule dynamics in eggs that did not contain F-actin—and thus experienced high incidence of chromatid scattering—significantly reduced both the extent of chromatid dispersion (Fig. 6, A and B; fig. S5, G and H; and movie S17) and speed of chromatid mobility (Fig. 6C and fig. S6D). These data collectively indicate that pulling forces mediated by microtubule dynamics—normally insufficient to fully disengage weakly bound chromatids—can cause complete chromatid separation when F-actin is lost (Fig. 6D).

## DISCUSSION

Our findings indicate that spindle F-actin disruption is a crucial factor in female reproductive aging that exacerbates aneuploidies arising from cohesion loss. Removal of F-actin in aged eggs with naturally reduced centromeric cohesion exacerbates premature chromatid separation. Consistently, experimentally reducing centromeric cohesion in young eggs only modestly causes untimely chromatid separation, unless F-actin is also disrupted. Because persistently misaligned chromatids in F-actin–disrupted eggs are frequently missegregated in anaphase II (*21*), we propose that aging-related F-actin dysfunction can explain the sudden rise in oocyte aneuploidy rate at advanced reproductive ages (*2*, *17*, *20*). This would be consistent with the spindle-specific physiological reduction of F-actin that we have identified in aged eggs.

**Fig. 7.**
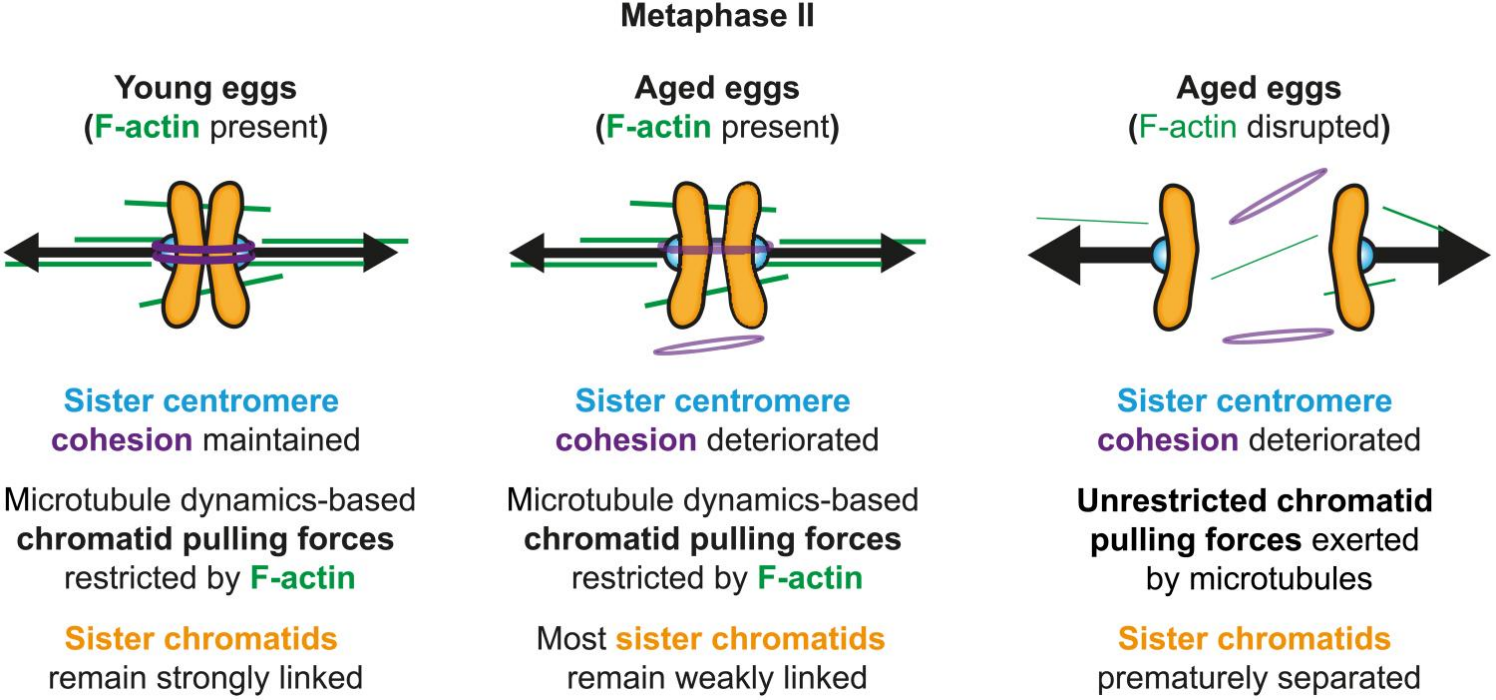
A model for a new function of F-actin in limiting aneuploidy during female reproductive aging. In young eggs containing full cohesin complement, F-actin reduces the effect of chromatid pulling forces, resulting in strong sister chromatid linkage. When cohesin is partially depleted in reproductive aging, F-actin–mediated resistance to microtubule pulling forces maintains linkage between most sister chromatids. When F-actin is disrupted in eggs with further reproductive aging, pulling forces generated by dynamic microtubules more effectively and prematurely separate sister chromatids. Aging-related F-actin disruption that follows cohesin depletion could therefore explain the marked rise in the incidence of egg aneuploidies near the end of female reproductive life.

Results from our combined cytoskeletal manipulation experiments indicate that F-actin exerts this previously unknown function by modulating microtubule dynamics. Because F-actin disruption in cohesion-deficient eggs causes extensive scattering of prematurely separated chromatids throughout the spindle, it is not possible to reproducibly measure microtubule dynamics parameters such as microtubule flux and turnover as we have done previously (*21*). However, our earlier findings showed that meiotic spindle F-actin stabilizes kinetochore-bound microtubules to promote accurate chromosome segregation (*21*). Furthermore, increasing the F-actin content of meiotic spindles substantially limits microtubule dynamics and reduces chromosomal separation in mouse oocytes (*21*). Our new data are therefore consistent with a model in which spindle-associated actin filaments generally dampen forces generated by dynamic microtubules (Fig. 7). In young eggs with strongly linked chromatids, this would supplement cohesion-mediated resistance to pulling forces exerted by dynamic microtubules, thus robustly generating interkinetochore tension. This force limitation would more critically resist microtubule-based chromatid pulling in aged eggs where cohesion is depleted (Fig. 7). This would explain why F-actin disruption in aged eggs leads to extensive separation of sister chromatids. Consistent with this model, female reproductive aging is accompanied by aberrant microtubule dynamics in mouse oocytes (*39*) that are proposed as a cohesion-independent cause of meiotic aneuploidy (*41*). Therefore, our findings also define F-actin disruption as a new link between cohesion deterioration and microtubule dysfunction in aging eggs. Unexpectedly, intercentromere distance measurements indicate that F-actin disruption predisposes only a subset of weakly associated chromatids to premature separation. Future experiments will address whether reproductive age-related dysfunction of spindle F-actin contributes to chromosome-specific egg aneuploidies, a source of chromosomal instability that is reported in somatic cells (*42*).

Unpaired chromatids in our female reproductive aging-like experimental system are generated during metaphase II arrest, after successful completion of anaphase I. Our high temporal live-imaging data show that shortly after their separation, single chromatids in control eggs are frequently repositioned to opposite sides of the spindle equator (fig. S4B and movie S6). By increasing the likelihood that these chromatids are subsequently segregated to opposite sides of the spindle at anaphase, these realignment events could reduce the likelihood of chromatid gain or loss during anaphase II. Our data show that in addition to causing unrestricted dispersion of prematurely separated chromatids, loss of F-actin also disrupts single chromatid realignment with some chromatids cycling between opposite sides of the spindle (movie S15). Together, these defects in chromosome dynamics are likely to increase chances of single chromatid missegregation in anaphase II. Directly visualizing whether chromatids that are prematurely separated in our experimental system become randomly misaligned at opposite sides of the metaphase II spindle and thus subsequently cause gain or loss of chromatids at anaphase II will require more precise tools for time-resolved degradation of Rec8 early in meiosis II. Innovation of live-imaging tools to directly visualize the dynamics of Rec8-containing cohesin complexes in young and aged oocytes will additionally inform how to best perform these cohesion depletion experiments. We previously showed that disruption of F-actin in meiosis I oocytes generates persistently misaligned chromosomes in meiosis II, which also randomly cycle throughout the spindle and are ultimately missegregated at anaphase II (*21*). Therefore, female reproductive aging-related disruption of spindle F-actin in oocytes likely amplifies centromeric cohesion loss-derived aneuploidy by randomizing the position of prematurely separated sister chromatids on the metaphase II spindle.

The actin cytoskeleton in mammalian oocytes has diverse functions beyond chromosome segregation (*43*). Although there are currently no experimental tools to specifically disrupt spindle-associated F-actin structures, we have here used pharmacological assays that we previously developed to systematically address the function of spindle F-actin in oocyte chromosome segregation (*21*, *22*). These experimental approaches have excluded contribution of cytoplasmic F-actin network organization and dynamics, actin-mediated vesicle trafficking, and cytokinesis to accurate chromosome segregation in oocytes. We therefore conclude that novel functions of the actin cytoskeleton we have uncovered here are specific to spindle-associated populations of F-actin. Reproductive age-associated depletion of spindle specific F-actin structures supports this conclusion. While these new functions are relevant to fundamental understanding of female meiosis, they could have important clinical implications, particularly whether F-actin restoration can be used to reduce aneuploidy rates in cohesion-deficient aged eggs. Reinforcing spindle F-actin by directly stabilizing it in aged eggs is unlikely to rescue aneuploidy as this approach can by itself induce severe chromosome segregation errors (*21*). Therefore, the next step is to understand the molecular causes of reproductive age-related spindle F-actin loss and to identify which actin- or microtubule-related proteins decline with aging. It can then be explored whether reintroduction of these spindle F-actin assembly proteins rescues accurate chromosome segregation in cohesion-deficient eggs obtained from reproductively older females.

F-actin and its associated proteins promote microtubule stability and organization in various cellular contexts, including in female meiosis (*21*, *44*–*51*). Moreover, recent findings show that F-actin is also a component of mitotic spindle machineries that separate sister chromatids (*52*, *53*). Our findings could therefore have broader implications for understanding the role of spindle F-actin in nonreproductive cells where aging is also associated with aneuploidies (*54*–*58*).

## MATERIALS AND METHODS

### Mouse oocyte isolation, maturation, culturing, and microinjection

All animal work in this research was performed at the University of Bristol and approved by the institution’s Animal Welfare and Ethical Review Body. Mice were maintained in a pathogen-free environment in accordance with U.K. Home Office regulations under the guidelines of the University of Bristol Animal Services Unit. Oocytes were isolated from the ovaries of 8- to 12-week CD1 or C57BL/6 (young) or 8 to 9 months CD1 or 13 to 14 months C57BL/6 (aged) mice, cultured, and microinjected with 6 to 8 pl of in vitro transcribed mRNA as described in detail recently (*22*). C57BL/6 mice were used for comparison of cytoplasmic and spindle F-actin populations between young and aged eggs as—owing to pandemic-associated supply chain failures—this was the only aged mouse strain commercially available to us in the early stages of the project.

### Cytoskeletal drug addition experiments

Metaphase II–arrested eggs (at least 4 hours after polar body extrusion) were treated for 4 hours with cytochalasin D (C8273-1MG, Merck) at a final concentration of 5 μg/ml in M2 medium or latrunculin B (428020-1MG, Merck) at a final concentration of 5 μM in M2 medium (to disrupt F-actin) and SiR-Actin (SC001, Spirochrome) at a final concentration of 10 μM in M2 medium (to stabilize F-actin). In our initial experiments, we used optimized concentrations of the microtubule-stabilizing compound Taxol to effectively block microtubule dynamics in mouse eggs. However, the combination of Taxol with cytochalasin D invariably disrupted meiotic spindles. We therefore used the docetaxel-derivative compound SiR-tubulin (SC002, Spirochrome) at a final concentration of 1 μM in M2 medium to stabilize microtubules. In simultaneous F-actin disruption and microtubule stabilization experiments, eggs were treated with SiR-tubulin (1 μM) for 2 hours and then with a combination cytochalasin D (5 μg/ml) and SiR-tubulin (1 μM) for 2 hours. In TRIM-Away experiments, drug treatment was performed before antibody microinjection. All drugs were dissolved in DMSO (D2650-5X5ML, Merck). In control conditions, DMSO was diluted in M2 medium identically to corresponding experimental conditions.

### Generation of expression constructs and mRNA synthesis

To label chromosomes, H2B-mRFP mRNA was transcribed from pGEM-H2B-mRP (*24*). To generate pGEM-SNAP-TRIM21 for TRIM-Away experiments, pGEM-N-SNAPf was first constructed by removing SNAPf from pSNAPf (New England Biolabs, N9183S) with Age I–Xho I and inserting it into the Age I–Xho I site of pGEM-HE (*59*). The coding sequence of mouse TRIM21 was then transferred from pGEM–enhanced green fluorescent protein–TRIM21 (*29*) into pGEM-N-SNAPf by Gibson assembly using primers 5′-TTAAACTCGAGCTCAAGCTTATGTCTCTGGAAAAGATG-3′ and 5′-ATCCCGGGCCCGCGGTACCGTCACATCTTTAGTGGACAG3-′.

Capped mRNAs were synthesized using a T7 polymerase (mMessage mMachine kit, Ambion). mRNA concentrations were measured using a NanoDrop spectrophotometer (Thermo Fisher Scientific).

### Fixation and immunostaining of mouse oocytes and eggs

Cells were fixed in 100 mM Hepes, 10 mM MgSO_4_, 50 mM EGTA, 0.5% Triton X-100 (v/v), and 2% (v/v) formaldehyde for 30 min at 37°C and then blocked at 4°C overnight in phosphate-buffered saline (PBS) containing 0.3% (v/v) Triton X-100 and 3% (w/v) bovine serum albumin (BSA). In Centromere protein A (CENP-A) immunostaining experiments, eggs were fixed at room temperature for 20 min, extracted in PBS containing 0.25% (v/v) Triton X-100 at room temperature for 10 min, and incubated in 3% (w/v) BSA-PBS at 4°C overnight. In Rec8 immunostaining staining experiments, the zona pellucida was removed before fixation by treating cells with Tyrode’s acidic solution (Merck, T1788-100ML) as described previously (*60*). In both CENP-A and Rec8 immunostaining experiments, fixed cells were incubated with λ-phosphatase (New England Biolabs, P0753S) for 2 hours at 30°C before immunostaining. Primary antibodies used were Rec8 rabbit antiserum [1:2000 dilution; a gift from M. Lampson produced as described previously (*19*)], CENP-A (1:200 dilution; Cell Signaling Technology, 2048S), topoisomerase II (1:200 dilution; Abcam, ab52934), and human anti-centromere antibody (1:200 dilution; Antibodies Incorporated, 15-234). Secondary antibodies and stains used were Alexa Fluor 488–labeled anti-rabbit (1:200 dilution; Molecular Probes), Alexa Fluor 488–phalloidin (1:20 dilution; Molecular Probes), and Hoechst 33342 (5 μg/ml; Molecular Probes).

### Metaphase chromosomal spreading, fixation, and immunostaining

To facilitate chromosomal spreading, the zona pellucida was removed from metaphase I oocytes or metaphase II–arrested eggs by washing cells through Tyrode’s acid solution (Merck, T1788-100ML) droplets covered with mineral oil (*60*). After washing out Tyrode’s solution in M2 medium, cells were recovered from acid treatment for a minimum of 5 min at 37°C. To spread chromosomes, two to three cells were dropped by pipetting onto a well of 15-well multitest slide (MP Biomedicals, 096041505) containing water-based spreading solution [1% (v/v) paraformaldehyde, 0.15% (v/v) Triton X-100, and 3 mM dithiothreitol (pH 9.2 to 9.4)] and air-dried in a nontransparent humidified box. After incubation with λ-phosphatase (New England Biolabs, P0753S) for 2 hours at 30°C, spreads were blocked in 3% (w/v) BSA (Thermo Fisher Scientific, 11483823) in PBS. Immunostaining was performed by washing out blocking solution in PBS, followed by sequential incubation with primary and secondary antibodies for 1.5 hours at 37°C. Primary antibodies were Rec8 rabbit antiserum [1:2000 dilution; as described above and produced previously (*19*)] and human anti-centromere antibody (1:2000 dilution; 15-234, Antibodies Incorporated). Secondary antibodies and stains used were Alexa Fluor 488–labeled anti-rabbit (1:2000 dilution; Molecular Probes), Alexa Fluor 488–labeled anti-human (1:2000 dilution; Molecular Probes), and Hoechst 33342 (5 μg/ml; Molecular Probes). Slides were prepared for microscopy by covering wells with VECTASHIELD Antifade Mounting Medium (2B Scientific, H-1000-10), mounting with 22 mm–by–22 mm glass coverslips (VWR, 631-0124), and sealing with nail varnish.

### High-resolution confocal live microscopy

Confocal time-lapse images of metaphase II–arrested mouse eggs were acquired using a Zeiss LSM 800 microscope equipped with an environmental chamber maintained at 37°C and a 40× C-Apochromat 1.2–numerical aperture (NA) water-immersion objective. Image acquisition using ZEN2 software (Zeiss) was performed at a temporal resolution of 5 min and with a z-stack thickness of ~40 μm at 1.5-μm confocal sections. Eggs were imaged in M2 medium (with or without cytoskeletal drugs) under mineral oil as described previously (*22*).

### Confocal, superresolution, and wide-field immunofluorescence microscopy

Confocal immunofluorescence images were acquired using a Zeiss LSM 800 confocal microscope, equipped with a 40× C-Apochromat 1.2-NA water immersion objective. z-stacks were acquired with a thickness of 2.5 μm at 0.5-μm confocal sections (for spindle microtubule fluorescence quantification), 15 μm at 0.3-μm confocal sections (for single chromatid quantification), or 30 μm at 0.5-μm confocal sections (for Rec8 fluorescence quantification).

For spindle F-actin imaging, superresolution 3D images of fluorescent phalloidin-labeled F-actin structures were acquired at the middle of the meiotic spindle in 0.5-μm steps over a range of 2.5 μm using the Airyscan module on a Zeiss LSM 800 microscope and a 40× C-Apochromat 1.2-NA water-immersion objective. Postacquisition superresolution images were obtained by 3D Airyscan processing of raw images in ZEN2 software (Zeiss).

For cytoplasmic F-actin imaging, single section superresolution images of fluorescent phalloidin-labeled F-actin structures were acquired at hemispheric regions of each egg using the Airyscan module on a Zeiss LSM 800 microscope and a 40× C-Apochromat 1.2-NA water-immersion objective.

3D immunofluorescence images of metaphase chromosomal spreads were acquired at 0.5-μm steps covering 5 μm using a Leica DMI6000 inverted wide-field microscope equipped with a 100× HCX PL APO CS oil-immersion objective.

Images in control and experimental conditions were acquired using identical imaging settings. Eggs were imaged in M2 medium under mineral oil as described previously (*22*).

### Fluorescence intensity quantification of Rec8

3D volumes of metaphase I chromosomes were reconstructed in Imaris software (Bitplane) using Hoechst 33342 immunofluorescence signal. Reconstructed surfaces were then used to mask chromosomes, remove background, and measure the mean fluorescence intensity of Rec8 on individual chromosomes.

Rec8 fluorescence intensity in metaphase I chromosomal spreads was quantified from sum intensity projections of z-stacks (ImageJ). Mean intensity measurements were performed in ImageJ by manually drawing a region of interest around each chromosome (fig. S3G). Mean fluorescence intensity measurements were normalized by dividing individual values in both control and experimental groups by the average of mean fluorescence intensity values in control groups.

### Quantification of cytoplasmic and spindle F-actin fluorescence intensities in young and aged mouse eggs

The ratio of spindle F-actin to cytoplasmic F-actin fluorescence intensity was determined from sum intensity projected z-stacks in ImageJ. Spindle F-actin fluorescence was measured by averaging the mean fluorescence intensities of five square regions of interest inside the spindle (fig. S1A). Cytoplasmic F-actin fluorescence was measured by averaging the mean fluorescence intensities of five similarly sized square regions of interest in the area immediately surrounding the spindle (fig. S1A). Ratios were obtained for each egg by dividing the average mean fluorescence intensity of spindle F-actin by the average mean fluorescence intensity of cytoplasmic F-actin.

Cytoplasmic F-actin fluorescence—not restricted to the region containing the meiotic spindle (fig. S1E)—was measured in ImageJ by averaging the mean fluorescence intensities of five square regions of interest inside each egg. Background removal was performed by subtracting the average mean fluorescence intensity of five similarly sized square regions of interest—placed outside the image of the cell and in regions that did not contain any phalloidin signal—from the averaged mean fluorescence intensity of cytoplasmic F-actin (fig. S1E). Mean fluorescence intensity measurements were normalized by dividing individual values in both control and experimental groups by the average of mean fluorescence intensity values in control groups.

### Quantification of spindle microtubule fluorescence intensity in young and aged mouse eggs

Spindle microtubule fluorescence was measured in ImageJ from sum intensity projected z-stacks by averaging the mean fluorescence intensities of five square regions of interest inside the spindle (fig. S1B). Background removal was performed by subtracting the average mean fluorescence intensity of five similarly sized square regions of interest—placed in cytoplasmic regions that did not contain any microtubule filament—from the averaged mean fluorescence intensity of spindle microtubules (fig. S1B). Mean fluorescence intensity measurements were normalized by dividing individual values in both control and experimental groups by the average of mean fluorescence intensity values in control groups.

### Quantification of prematurely separated sister chromatids and intercentromere distances

Chromatids in metaphase II–arrested eggs and metaphase II chromosomal spreads were identified by reconstructing the 3D volume of topoisomerase II immunofluorescence signal (eggs) or Hoechst fluorescence signal (spreads) in Imaris software (Bitplane). Centromeres were then identified using CENP-A immunofluorescence signal for spot detection in Imaris. Last, neighboring centromeres were manually assigned as sisters using distance measurements between sister centromeres and reconstructed chromatid volumes as reference. Chromatids containing centromeres for which no corresponding sisters were identified via this analysis were designated as prematurely separated chromatids (Fig. 1C). Single chromatids in metaphase II spreads were identified via Hoechst labeling and centromere distance measurements in combination with spot detection in Imaris using the immunofluorescence signal of human anticentromere antibody.

### Partial and complete targeted degradation of Rec8 in TRIM-Away experiments

In partial Rec8 degradation TRIM-Away experiments, TRIM21 expressing, metaphase II–arrested eggs were microinjected with 2 to 3 pl of Rec8 antiserum (1:30 to 1:50 dilution) (*19*) and Alexa Fluor 488 Dextran 10,000 molecular weight (MW) (to validate antibody microinjection by fluorescence imaging) (1:40 dilution; Molecular Probes, D22910) in 0.05% (v/v) NP-40–PBS. In complete Rec8 degradation TRIM-Away experiments, TRIM21-expressing, metaphase II–arrested eggs were microinjected with 2 to 3 pl of Rec8 antiserum (1:2 dilution) (*19*) and Alexa Fluor 488 Dextran 10,000 MW (1:40 dilution; Molecular Probes, D22910) in 0.05% (v/v) NP-40–PBS. In simultaneous Rec8 degradation and cytoskeletal manipulation experiments, all TRIM-Away microinjections were performed in M2 medium containing individual or a combination of cytoskeletal drugs as appropriate.

### 3D chromatid surface reconstruction and scattering volume measurement

Chromatid surfaces were reconstructed in 3D in Imaris (Bitplane) from high-resolution time-lapse movies of H2B-mRFP. Object-oriented bounding box analysis was performed in Imaris to identify the minimal cuboid volume that enclosed all chromatids at each time point. This bounding box volume was defined as the chromatid scattering volume. For each egg, data normalization was performed by dividing measured values at each time point with the bounding box volume at the start of the live-imaging experiment (*t* = 0 min).

### Quantification of 3D chromatid movement speed and realignment

To quantify chromatid mobility, the 3D volumes of chromatids were reconstructed in Imaris (Bitplane) from high-resolution live-imaging datasets of H2B-mRFP. For chromatids that distinctly migrated away from the spindle equator in control and experimental conditions, frame-to-frame displacement was measured by tracking the position of chromatid, leading ends via the measurement points function in Imaris. Displacement values (micrometers) were divided by the temporal resolution of live-imaging experiments (5 min) to calculate instantaneous chromatid movement speeds.

Realignment of previously scattered single chromatids to the spindle equator was analyzed in 3D in Imaris. A realignment event was defined as the merging of a misaligned chromatid with the main chromosome mass for a duration of at least 10 min.

### Statistical data analyses

Histograms, box plots, and other graphs were generated using OriginPro (OriginLab) or Prism (GraphPad) software. Box plots show mean (line), 5th, 95th (whiskers), 25th, and 75th percentiles (box enclosing 50% of the data) and are overlaid with individual data points. Statistical significance evaluations and one-way and two-way analyses of variance (ANOVAs) were performed in OriginPro or Prism software. Significance values are *P < 0.05, **P < 0.005, and ***P < 0.0005. Nonsignificant values are indicated as N.S.
