# Supplementary material for: Actin limits egg aneuploidies associated with female reproductive aging
Source: Sci Adv. 2023 Jan 20;9(3):eadc9161. doi: 10.1126/sciadv.adc9161 (PMC9858517; doi:10.1126/sciadv.adc9161)
Supplement: Supplementary file 1 — Figs. S1 to S6 [file sciadv.adc9161_sm.pdf]

Science Advances
AAAS

Supplementary Materials for

Actin limits egg aneuploidies associated with female reproductive aging

Sam Dunkley and Binyam Mogessie

Corresponding author: Binyam Mogessie, binyam.mogessie@yale.edu

Sci. Adv. **9**, eadc9161 (2023)
DOI: 10.1126/sciadv.adc9161

The PDF file includes:

Figs. S1 to S6
Legends for movies S1 to S17

Other Supplementary Material for this manuscript includes the following:

Movies S1 to S17

Fig. S1

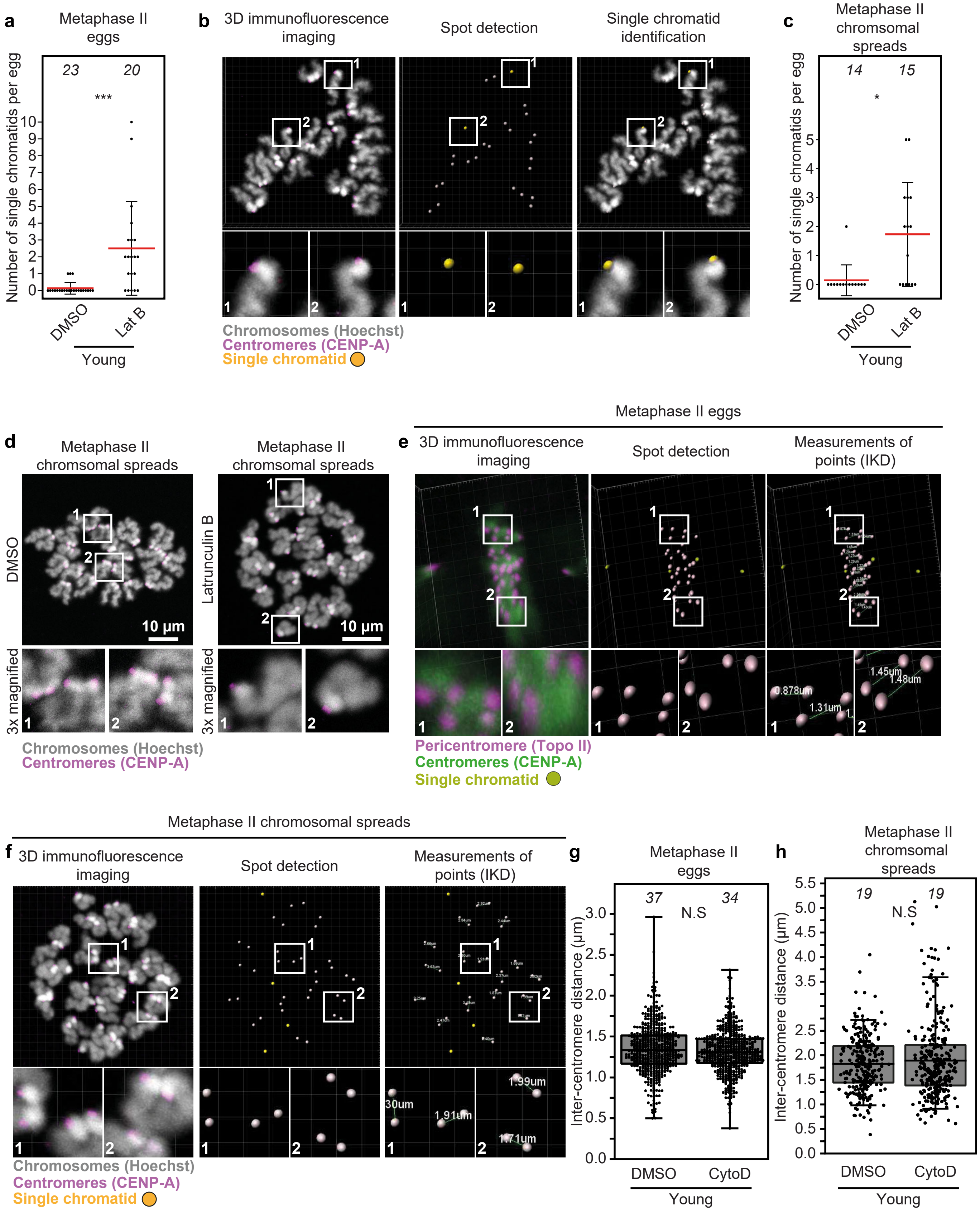
a
Metaphase II eggs
Number of single chromatids per egg
23
20
***
DMSO
Lat B
Young
b
3D immunofluorescence imaging
Spot detection
Single chromatid identification
Chromosomes (Hoechst)
Centromeres (CENP-A)
Single chromatid
c
Metaphase II chromsomal spreads
Number of single chromatids per egg
14
15
*
DMSO
Lat B
Young
d
Metaphase II chromsomal spreads
DMSO
Latrunculin B
10 µm
3x magnified
Chromosomes (Hoechst)
Centromeres (CENP-A)
Metaphase II eggs
e
3D immunofluorescence imaging
Spot detection
Measurements of points (IKD)
0.878um
1.31um
1.45um
1.48um
Pericentromere (Topo II)
Centromeres (CENP-A)
Single chromatid
Metaphase II chromosomal spreads
f
3D immunofluorescence imaging
Spot detection
Measurements of points (IKD)
1.99um
1.91um
1.71um
Chromosomes (Hoechst)
Centromeres (CENP-A)
Single chromatid
g
Metaphase II eggs
Inter-centromere distance (µm)
37
34
N.S
DMSO
CytoD
Young
h
Metaphase II chromsomal spreads
Inter-centromere distance (µm)
19
19
N.S
DMSO
CytoD
Young

Fig. S1. F-actin loss predisposes eggs to aging-like premature chromatid separation

(a) Quantification of the number of single chromatids (identified as in Fig. 2a) in DMSO- or Latrunculin B-treated metaphase II-arrested eggs. Each filled black circle in graph represents a single egg and red bars represent mean values. Data are from 3 independent experiments.

(b) High-resolution microscopy pipeline (described in materials and methods section) for identification and quantification of prematurely separated centromeres in metaphase II chromosomal spreads.

(c) Quantification of the number of single chromatids in metaphase II chromosomal spreads of DMSO- or Latrunculin B-treated eggs. Each filled black circle in graph represents chromosomal spread from a single egg and red bars represent mean values. Data are from 3 independent experiments.

(d) Representative maximum intensity projected immunofluorescence images of centromeres and chromatids in metaphase II chromosomal spreads of DMSO- or Latrunculin B-treated young eggs. Boxes mark regions that are magnified in insets.

(e) High-resolution microscopy pipeline (described in materials and methods section) for measurement of inter-centromere distances in metaphase II-arrested eggs.

(f) High-resolution microscopy pipeline (described in materials and methods section) for measurement of inter-centromere distances in metaphase II chromosomal spreads.

(g) Distribution of inter-centromere distances (measured as in Fig. S1e) in DMSO- or Cytochalasin D-treated metaphase II-arrested young eggs. Data are from 3 independent experiments.

(h) Distribution of inter-centromere distances (measured as in Fig. S1f) in DMSO- or Cytochalasin D-treated metaphase II-arrested young eggs. Data are from 3 independent experiments.

Statistical significance was evaluated using Mann-Whitney *t*-test (a and c) or two-tailed Student's t-test (g and h). The number of analyzed oocytes is specified in italics.

Fig. S2

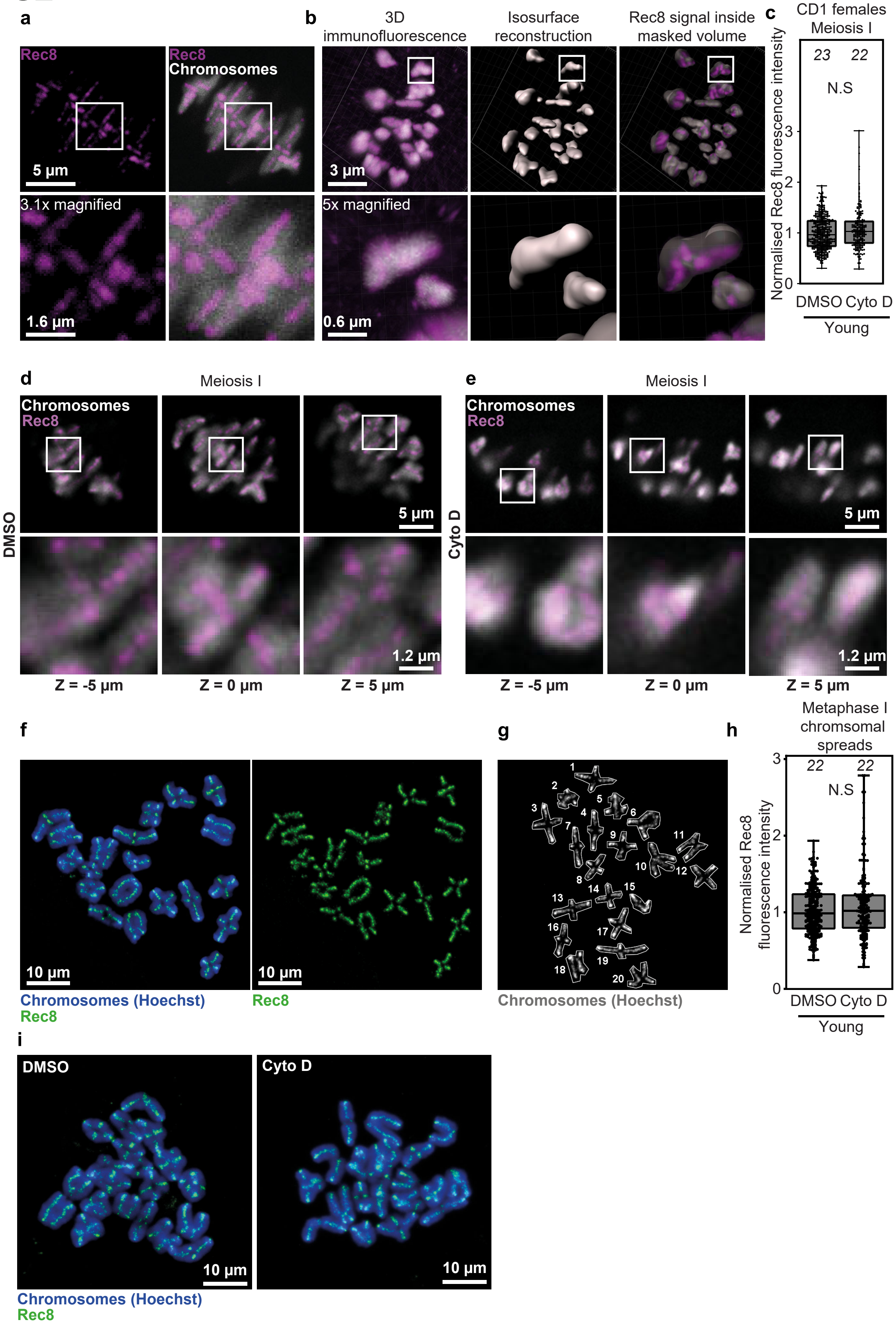
a
Rec8
Rec8
Chromosomes
5 µm
3.1x magnified
1.6 µm
b
3D immunofluorescence
Isosurface reconstruction
Rec8 signal inside masked volume
3 µm
5x magnified
0.6 µm
c
CD1 females Meiosis I
23
22
N.S
Normalised Rec8 fluorescence intensity
0
1
2
3
DMSO
Cyto D
Young
d
Meiosis I
Chromosomes
Rec8
DMSO
5 µm
1.2 µm
Z = -5 µm
Z = 0 µm
Z = 5 µm
e
Meiosis I
Chromosomes
Rec8
Cyto D
5 µm
1.2 µm
Z = -5 µm
Z = 0 µm
Z = 5 µm
f
10 µm
10 µm
Chromosomes (Hoechst)
Rec8
Rec8
g
1 2 3 4 5 6 7 8 9 10 11 12 13 14 15 16 17 18 19 20
Chromosomes (Hoechst)
h
Metaphase I chromsomal spreads
22
22
N.S
Normalised Rec8 fluorescence intensity
0
1
2
3
DMSO
Cyto D
Young
i
DMSO
Cyto D
10 µm
10 µm
Chromosomes (Hoechst)
Rec8

Fig. S2. F-actin disruption does not impact centromeric Rec8 cohesin complexes

(a) Maximum intensity projected high-resolution immunofluorescence images of Rec8 cohesin complexes and homologous chromosomes in a mouse oocyte. Boxes mark regions that are magnified in insets.

(b) Rec8 immunofluorescence intensity quantification pipeline. Individual chromosome volumes were reconstructed using the Surfaces module of Imaris. Mean Rec8 fluorescence intensity was then measured inside the masked volume each chromosome.

(c) Normalized Rec8 mean fluorescence intensities in DMSO- or Cytochalasin D-treated mouse oocytes. Data are from 3 independent experiments.

(d) Representative single confocal section immunofluorescence images of Rec8 and chromosomes – spaced 5 μm apart – in DMSO-treated mouse oocytes. Boxes mark regions that are magnified in insets.

(e) Representative single confocal section immunofluorescence images of Rec8 and chromosomes – spaced 5 μm apart – in Cytochalasin D-treated mouse oocytes. Boxes mark regions that are magnified in insets.

(f) Representative maximum intensity projected immunofluorescence images of Rec8 and chromosomes in metaphase I chromosomal spread of a mouse oocyte.

(g) Method (described in Materials and Methods) for quantification of Rec8 mean fluorescence intensity in metaphase I chromosomal spreads of mouse oocytes.

(h) Normalized Rec8 mean fluorescence intensities in metaphase I chromosomal spreads of DMSO- or Cytochalasin D-treated mouse oocytes. Data are from 3 independent experiments.

(i) Representative maximum intensity projected immunofluorescence images of Rec8 and chromosomes in metaphase I chromosomal spreads of DMSO- or Cytochalasin D-treated mouse oocytes.

Statistical significance was evaluated using Mann-Whitney (c) and Welch's (h) *t*-test. The number of analyzed oocytes is specified in italics.

Fig. S3

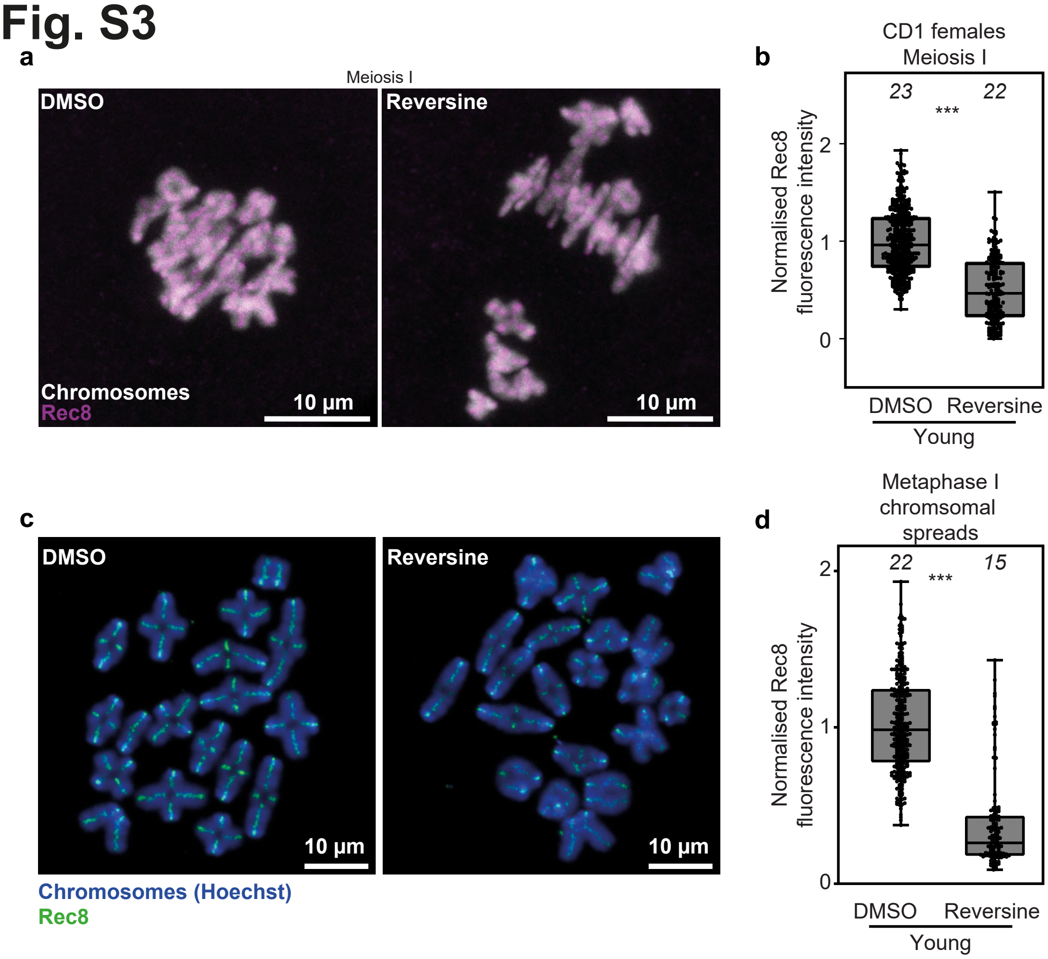
a
Meiosis I
DMSO
Reversine
Chromosomes
Rec8
10 µm
10 µm
b
CD1 females
Meiosis I
23
22
***
Normalised Rec8 fluorescence intensity
0
1
2
DMSO
Reversine
Young
c
DMSO
Reversine
10 µm
10 µm
Chromosomes (Hoechst)
Rec8
d
Metaphase I chromsomal spreads
22
15
***
Normalised Rec8 fluorescence intensity
0
1
2
DMSO
Reversine
Young

Fig. S3. MPS1 kinase inhibition reduces Rec8 cohesin complexes at the centromere.

(a) Maximum intensity projected high-resolution immunofluorescence images of Rec8 cohesin complexes and homologous chromosomes in DMSO- or Reversine-treated mouse oocytes.

(b) Normalized Rec8 mean fluorescence intensities in metaphase I chromosomal spreads of DMSO- or Reversine-treated mouse oocytes. Data are from 3 independent experiments.

(c) Representative maximum intensity projected immunofluorescence images of Rec8 and chromosomes in metaphase I chromosomal spreads of DMSO- or Reversine-treated mouse oocytes.

(d) Normalized Rec8 mean fluorescence intensities in metaphase I chromosomal spreads of DMSO- or Reversine-treated mouse oocytes. Data are from 3 independent experiments.

Statistical significance was evaluated using Mann-Whitney test. The number of analyzed oocytes is specified in italics.

Fig. S4

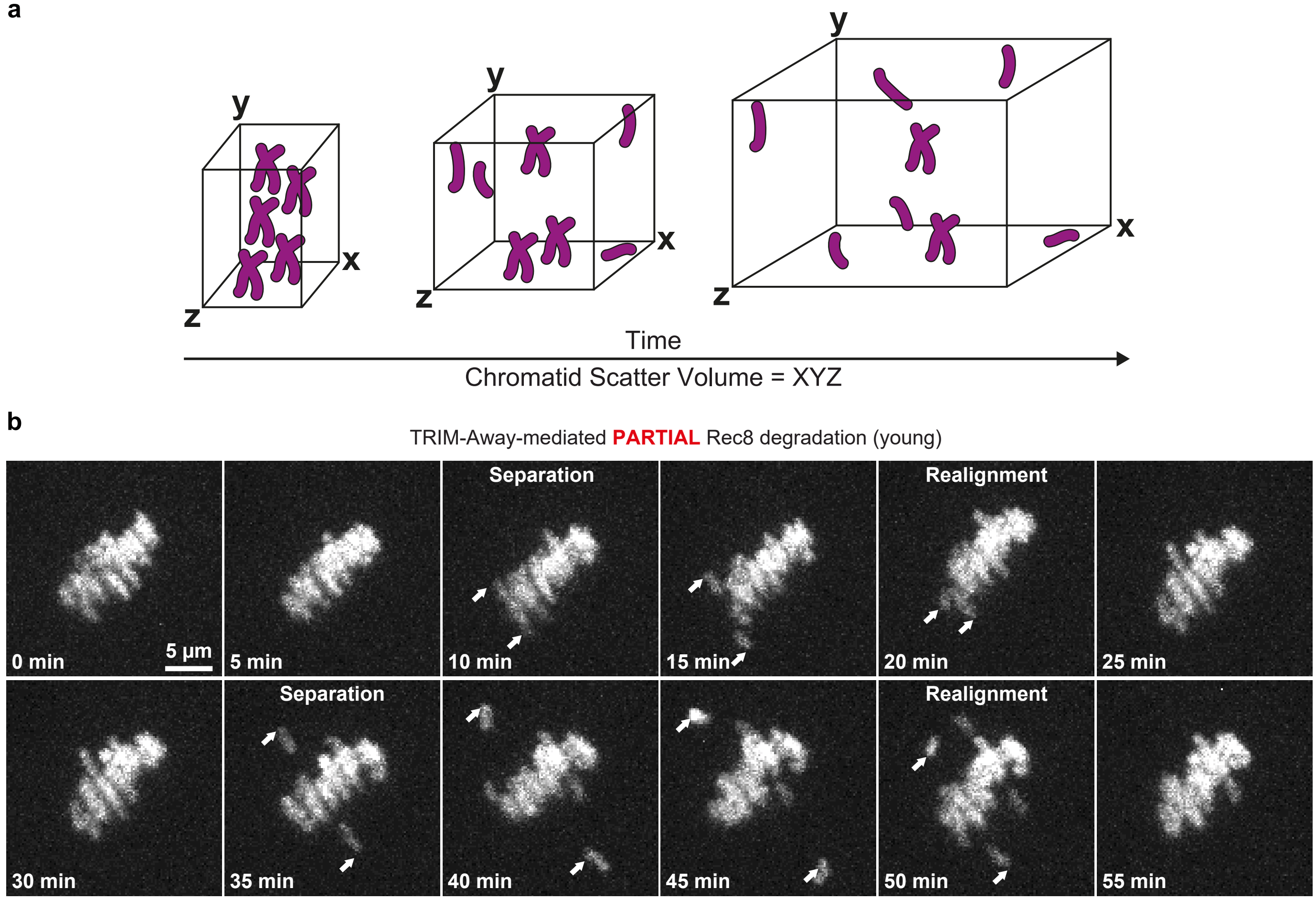
a
y
x
z
Time
Chromatid Scatter Volume = XYZ
b
TRIM-Away-mediated PARTIAL Rec8 degradation (young)
0 min
5 µm
5 min
Separation
10 min
15 min
Realignment
20 min
25 min
30 min
Separation
35 min
40 min
45 min
Realignment
50 min
55 min

Fig. S4 Quantitative live imaging pipeline for analysis of premature chromatid separation.

(a) Schematics of object-oriented bounding box analysis to measure the minimal cuboid volume that contains all chromatids at each live imaging timepoint. We used this analysis to obtain the scattering volume of prematurely separated chromatids in metaphase II-arrested mouse eggs.

(b) Representative maximum intensity projected high-resolution confocal images of sister chromatids in a metaphase II-arrested mouse egg with partially degraded Rec8. Arrows indicate modest chromatid separation and subsequent realignment events.

Fig. S5

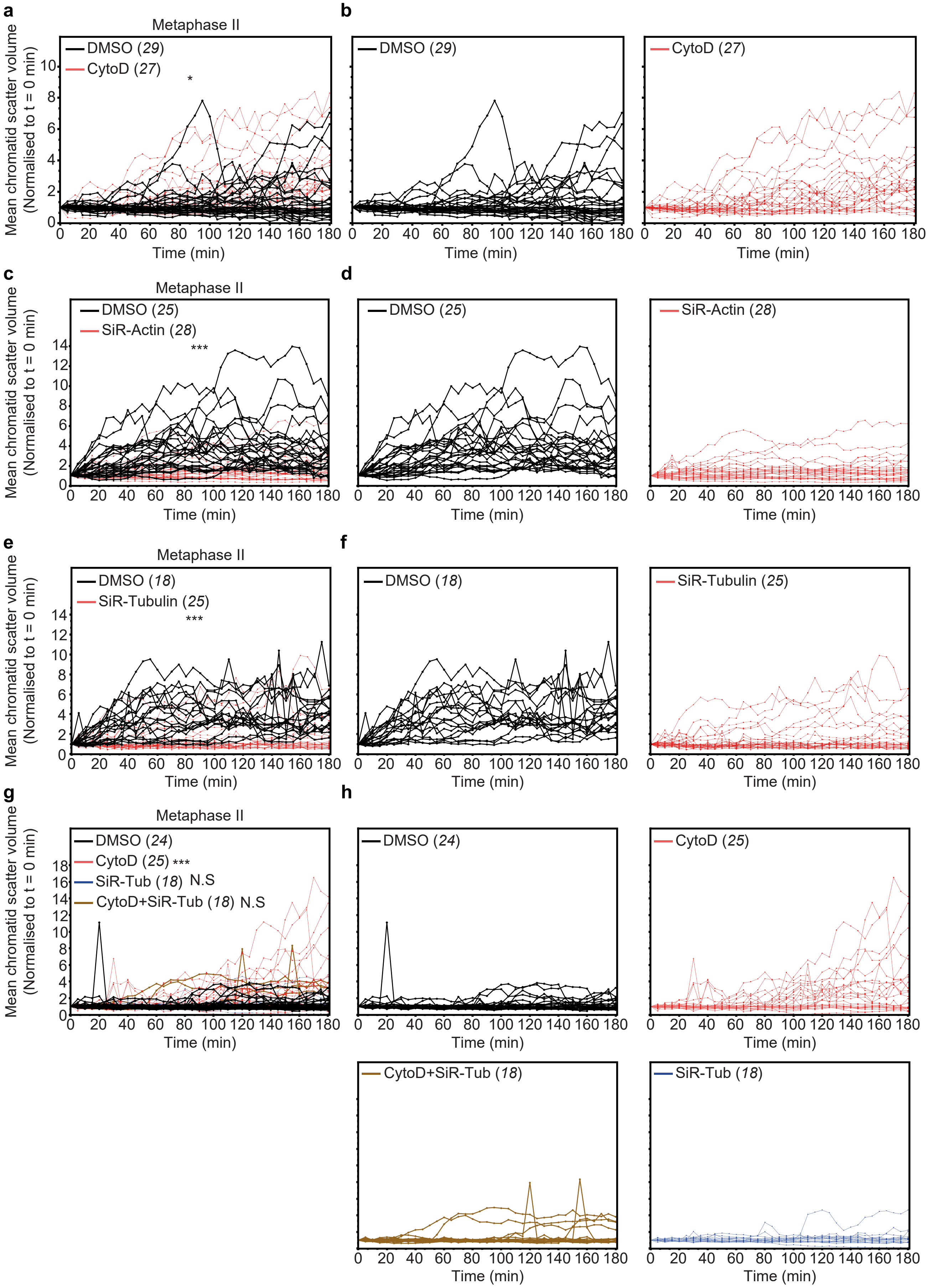

a
Metaphase II
DMSO (29)
CytoD (27)
*
Mean chromatid scatter volume
(Normalised to t = 0 min)
Time (min)
b
DMSO (29)
CytoD (27)
c
Metaphase II
DMSO (25)
SiR-Actin (28)
***
d
DMSO (25)
SiR-Actin (28)
e
Metaphase II
DMSO (18)
SiR-Tubulin (25)
***
f
DMSO (18)
SiR-Tubulin (25)
g
Metaphase II
DMSO (24)
CytoD (25) ***
SiR-Tub (18) N.S
CytoD+SiR-Tub (18) N.S
h
DMSO (24)
CytoD (25)
CytoD+SiR-Tub (18)
SiR-Tub (18)

Fig. S5. F-actin limits aging-like premature chromatid separation by microtubule-based pulling forces.

(a) Normalized chromatid scatter volumes measured as in Figs. 3b and S4a over 3 hours in DMSO- or Cytochalasin D-treated metaphase II-arrested eggs with partially degraded Rec8. Each black and red line represents measurements from a single egg. Averaged measurements are presented in Fig. 3d. Data are from 3 independent experiments.

(b) Individual plots of normalized chromatid scatter volume data from (a) in DMSO- (black lines) or Cytochalasin D-treated (red lines) metaphase II-arrested eggs with partially degraded Rec8. Each line represents measurements from a single egg.

(c) Normalized chromatid scatter volumes measured as in Figs. 3b and S4a over 3 hours in DMSO- or SiR-Actin-treated metaphase II-arrested eggs with fully degraded Rec8. Each black and red line represents measurements from a single egg. Averaged measurements are presented in Fig. 4c. Data are from 3 independent experiments.

(d) Individual plots of normalized chromatid scatter volume data from (a) in DMSO- (black lines) or SiR-Actin-treated (red lines) metaphase II-arrested eggs with fully degraded Rec8. Each line represents measurements from a single egg.

(e) Normalized chromatid scatter volumes measured as in Figs. 3b and S4a over 3 hours in DMSO- or SiR-Tubulin-treated metaphase II-arrested eggs with fully degraded Rec8. Each black and red line represents measurements from a single egg. Averaged measurements are presented in Fig. 5b. Data are from 3 independent experiments.

(f) Individual plots of normalized chromatid scatter volume data from (a) in DMSO- (black lines) or SiR-Tubulin-treated (red lines) metaphase II-arrested eggs with fully degraded Rec8. Each line represents measurements from a single egg.

(g) Normalized chromatid scatter volumes measured as in Figs. 3b and S4a over 3 hours in DMSO-, Cytochalasin D-, SiR-Tubulin- or Cytochalasin D and SiR-Tubulin-treated metaphase II-arrested eggs with partially degraded Rec8. Each black and red line represents measurements from a single egg. Averaged measurements are presented in Fig. 6b. Data are from 3 independent experiments.

(h) Individual plots of normalized chromatid scatter volume data from (a) in DMSO- (black lines), Cytochalasin D- (red lines), SiR-Tubulin- (blue lines) or Cytochalasin D and SiR-

Tubulin-treated (brown lines) metaphase II-arrested eggs with partially degraded Rec8. Each line represents measurements from a single egg.

Statistical significance was evaluated using two-way ANOVA. The number of analyzed oocytes is specified in brackets and in italics.

Fig. S6

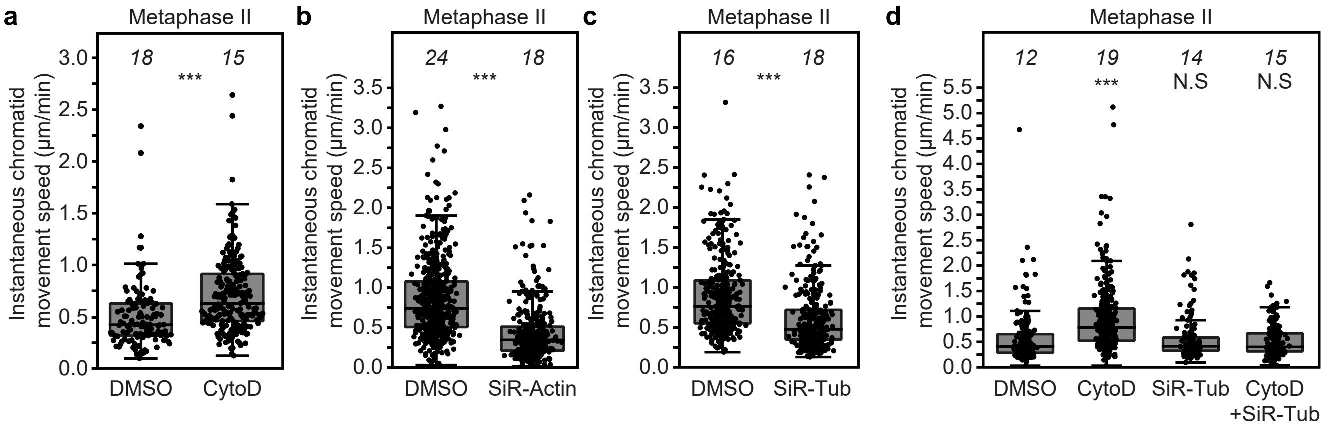
a
Metaphase II
Instantaneous chromatid movement speed (µm/min)
18
15
***
DMSO
CytoD
b
Metaphase II
24
18
***
DMSO
SiR-Actin
c
Metaphase II
16
18
***
DMSO
SiR-Tub
d
Metaphase II
12
19
14
15
***
N.S
N.S
DMSO
CytoD
SiR-Tub
CytoD +SiR-Tub

Fig. S6. F-actin loss accelerates chromatid separation following cohesion weakening in a microtubule dynamics-dependent manner

(a) Distribution of instantaneous chromatid movement speeds in DMSO- or Cytochalasin D-treated metaphase II-arrested eggs with partially degraded Rec8. Data are from 3 independent experiments and are also presented as relative frequency histograms in Fig. 3e.

(b) Distribution of instantaneous chromatid movement speeds in DMSO- or SiR-Actin-treated metaphase II-arrested eggs with partially degraded Rec8. Data are from 3 independent experiments and are also presented as relative frequency histograms in Fig. 4d.

(c) Distribution of instantaneous chromatid movement speeds in DMSO- or SiR-Tubulin-treated metaphase II-arrested eggs with partially degraded Rec8. Data are from 3 independent experiments and are also presented as relative frequency histograms in Fig. 5c.

(d) Distribution of instantaneous chromatid movement speeds in DMSO-, Cytochalasin D-, SiR-Tubulin- or Cytochalasin D and SiR-Tubulin-treated metaphase II-arrested eggs with partially degraded Rec8. Data are from 3 independent experiments and are also presented as relative frequency histograms in Fig. 6c.

Statistical significance was evaluated using two-way ANOVA. The number of analyzed oocytes is specified in italics.

Movie S1. Navigation through single immunofluorescence confocal sections – spaced 0.5 μm apart – of Rec8 (magenta) and homologous chromosomes (grey) in a DMSO-treated mouse oocyte.

Movie S2. Navigation through single immunofluorescence confocal sections – spaced 0.5 μm apart – of Rec8 (magenta) and homologous chromosomes (grey) in a Cytochalasin D-treated mouse oocyte.

Movie S3. Time lapse movie of modest separation of chromatids (H2B-mRFP, magenta) in a metaphase II-arrested mouse egg with partially degraded Rec8.

Movie S4. 3D isosurface reconstruction (Imaris) of chromatids in movie 3 for bounding box analysis of chromatid scattering.

Movie S5. Time lapse movie of modest separation of chromatids (H2B-mRFP, magenta) in a DMSO-treated metaphase II-arrested mouse egg with partially degraded Rec8.

Movie S6. Time lapse movie of modest separation and subsequent realignment (indicated by write arrows) of chromatids (H2B-mRFP, magenta) in a DMSO-treated metaphase II-arrested mouse egg with partially degraded Rec8.

Movie S7. Time lapse movie of excessive separation and scattering of chromatids (H2B-mRFP, magenta) in a Cytochalasin D-treated metaphase II-arrested mouse egg with partially degraded Rec8 (example 1).

Movie S8. Time lapse movie of excessive separation and scattering of chromatids (H2B-mRFP, magenta) in a Cytochalasin D-treated metaphase II-arrested mouse egg with partially degraded Rec8 (example 2).

Movie S9. Time lapse movie of complete separation and scattering of chromatids (H2B-mRFP, magenta) in a metaphase II-arrested mouse egg with fully degraded Rec8.

Movie S10. Time lapse movie of complete separation and scattering of chromatids (H2B-mRFP, magenta) in a DMSO-treated (control for SiR-Actin treatment) metaphase II-arrested mouse egg with fully degraded Rec8.

Movie S11. Time lapse movie of restricted movement of chromatids (H2B-mRFP, magenta) in a SiR-Actin-treated metaphase II-arrested mouse egg with fully degraded Rec8. SiR-Actin fluorescence in shown in grey.

Movie S12. Time lapse movie of complete separation and scattering of chromatids (H2B-mRFP, magenta) in a DMSO-treated (control for SiR-Tubulin treatment) metaphase II-arrested mouse egg with fully degraded Rec8.

Movie S13. Time lapse movie of restricted movement of chromatids (H2B-mRFP, magenta) in a SiR-Tubulin-treated metaphase II-arrested mouse egg with fully degraded Rec8. SiR-Tubulin fluorescence in shown in green.

Movie S14. Time lapse movie of complete separation and scattering of chromatids (H2B-mRFP, magenta) in a DMSO-treated (control for Cytochalasin D + SiR-Tubulin treatment) metaphase II-arrested mouse egg with partially degraded Rec8.

Movie S15. Time lapse movie of excessive separation and scattering of chromatids (H2B-mRFP, magenta) in a Cytochalasin D-treated (control for Cytochalasin D + SiR-Tubulin treatment) metaphase II-arrested mouse egg with partially degraded Rec8.

Movie S16. Time lapse movie of restricted movement of chromatids (H2B-mRFP, magenta) in a SiR-Tubulin-treated (control for Cytochalasin D + SiR-Tubulin treatment) metaphase II-arrested mouse egg with partially degraded Rec8. SiR-Tubulin fluorescence in shown in green.

Movie S17. Time lapse movie of restricted movement of chromatids (H2B-mRFP, magenta) in a Cytochalasin D + SiR-Tubulin-treated metaphase II-arrested mouse egg with partially degraded Rec8. SiR-Tubulin fluorescence in shown in green.
